# Construction of Fluorescent Conjugated Polytriazole Containing Double-Decker Silsesquioxane: Click Polymerization and Thermal Stability

**DOI:** 10.3390/polym15020331

**Published:** 2023-01-09

**Authors:** Chia-Husan Chiang, Mohamed Gamal Mohamed, Wei-Cheng Chen, Manivannan Madhu, Wei-Lung Tseng, Shiao-Wei Kuo

**Affiliations:** 1 Department of Materials and Optoelectronic Science, College of Semiconductor and Advanced Technology Research, Center for Functional Polymers and Supramolecular Materials, National Sun Yat-sen University, Kaohsiung 804, Taiwan; 2 Chemistry Department, Faculty of Science, Assiut University, Assiut 71515, Egypt; 3 Department of Chemistry, National Sun Yat-sen University, Kaohsiung 804, Taiwan; 4 Department of Medicinal and Applied Chemistry, Kaohsiung Medical University, Kaohsiung 807, Taiwan

**Keywords:** double-decker silsesquioxane, polytriazole, click polymerization, thermal stability

## Abstract

This study synthesized two azide-functionalized monomers through *p*-dichloro xylene and double-decker silsesquioxane (DDSQ) units with NaN_3_ to form DB-N_3_ and DDSQ-N_3_ monomers, respectively. In addition, five different propargyl-functionalized monomers were also prepared from hydroquinone, bisphenol A, bis(4-hydroxyphenyl)methanone, 2,4-dihydroxybenzaldehyde (then reacted with hydrazine hydrate solution) and 1,2-bis(4-hydroxyphenyl)-1,2-diphenylethene with propargyl bromide to form P-B, P-BPA, P-CO, P-NP, and P-TPE monomers, respectively. As a result, various DDSQ-based main chain copolymers could be synthesized using Cu(I)-catalyzed click polymerization through DDSQ-N_3_ with different propargyl-functionalized monomers, of which the chemical structure and molecular weight could be confirmed by using Fourier-transform infrared spectroscopy (FTIR), nuclear magnetic resonance (NMR), and gel permeation chromatography (GPC) analyses. Differential scanning calorimetry (DSC), thermogravimetric analysis (TGA), scanning electron microscope (SEM), transmission electron microscopy (TEM), and photoluminescence (PL) spectroscopy analyses also could characterize the thermal stability, morphology, and optical behaviors of these DDSQ-based copolymers. All results indicate that the incorporation of an inorganic DDSQ cage could improve the thermal stability such as thermal decomposition temperature and char yield, because of the DDSQ dispersion homogeneously in the copolymer matrix, and this would then affect the optical properties of NP and TPE units in this work.

## 1. Introduction

Forms of silsesquioxane (SiO_1.5_)_n_ as the main structure of siloxane can be divided into random, ladder, and polyhedrons according to their structure [1,2,3,4,5,6,7,8,9,10]. The polyhedron structures include complete cage and partial cage structures [11,12,13,14,15,16,17,18,19,20]. Polyhedral oligomeric silsesquioxane (POSS) as an inorganic cage structure in the polymer matrix could improve the thermal, mechanical, surface, and dielectric properties that have been widely investigated during the last two decades [21,22,23,24,25] because of its high molecular weight and three-dimensional structure that could reduce the possibility of molecular stacking and even crystallization. Our previous studies have proposed the polymer matrix’s non-functional, mono-functional, di-functional, and multi-functional POSS cage structures [15]. In general, the miscibility behavior is not good for non-functional POSS cages in the polymer matrix without specific interaction. Thus, the mono-, di-, or multi-functional POSS cage structures were synthesized using a covalent bond connected to the polymer chain to avoid the macro-phase separation of the POSS cage. Compared with a mono-functional POSS cage as the chain end or side chain into the polymer matrix [26,27,28,29,30], the multi-functional POSS cage would form a network structure that could not dissolve in common solvents, which would restrict the potential applications for future processing [31,32,33,34,35]. As a result, the preparation of di-functional POSS mostly adopts the *T*_10_ type to prepare double-decker silsesquioxanes (DDSQs) into the polymer matrix as the bead-type main chain of polymer/POSS hybrids have also been investigated recently [36,37,38,39,40]. DDSQ is the silsesquioxane featuring two reactive end groups on the diagonal position. These reactive end groups could be homogeneously dispersed and connected into a polymer network through a covalent bond [41,42,43,44]. Similar to the typical POSS cage, DDSQ-based hybrid copolymers could also improve thermal stability, and have low dielectric constant, mechanical properties, and excellent chain flexibility. The tethered hydroxyl, phenolic, amine, or other functionalized units could be synthesized through a hydrosilylation reaction using a vinyl-functionalized monomer with DDSQ under a Pt catalyst. However, the isomers would co-exist from α and β-hydrosilylation in these DDSQ-derivatives, which would affect the DDSQ packing in the main chain type of DDSQ-based copolymers to influence the thermal and mechanical properties as expected [1,3,6]. Therefore, in this study, we used another approach to prepare the di-functionalized chloride DDSQ without α and β-hydrosilylation through double-decker silsesquioxane-Na with dichloro(chloromethyl)methylsilane and then reacted this with NaN_3_ to form a di-functionalized azide DDSQ (DDSQ-N_3_) monomer. These azide units of DDSQ could perform the typical Cu(I)-catalyzed click polymerization with various propargyl-functionalized monomers, including P-B, P-BPA, P-CO, P-NP, and P-TPE monomers, to form the main chain type of DDSQ-based copolymers. The chemical structure and molecular weight of these could be confirmed by using FTIR, NMR, and GPC analyses. The thermal stability, morphology, and optical behaviors of these DDSQ-based copolymers could be characterized using DSC, TGA, SEM, TEM, and PL spectroscopy analyses.

## 2. Materials and Methods

### 2.1. Materials

4-Hydroxybenzophenone, zinc powder, potassium carbonate (K_2_CO_3_), titanium tetrachloride (TiCl_4_), propargyl bromide, acetonitrile, sodium azide (NaN_3_), pentamethyldiethylnetriamine (PMDETA), *p*-dichloro-xylene, CuBr, 2,4-dihydroxybenzaldehyde (BZ-2OH), tetrahydrofuran (THF), ethyl acetate (EA), acetone, dichloromethane (DCM), hexane, methanol (CH_3_OH), N, N-dimethylformamide (DMF), and hydrazine hydrate solution (NH_2_NH_2_.H_2_O) were acquired from J.T. Baker. Phenyltrimethylsilane, dichloro(chloromethyl)methylsilane, sodium hydroxide (NaOH), and 2-propanol were purchased from Sigma-Aldrich. Double-decker silsesquioxane-Na (DD-Na) was synthesized according to a previous procedure [22,45,46,47].

### 2.2. Synthesis of Double-Decker Silsesquioxane-Cl (DDSQ-Cl)

DD-Na (10 g, 8.62 mmol), triethylamine (3.59 g, 34.48 mmol), and THF (100 mL) were put in a three-necked flask, stirred thoroughly at room temperature, and then dichloro(chloromethyl) methylsilane (2.79 g, 17.2 mmol) was added as an end-capping agent. After 12 h, the filtrate of the mixture was purified with NaHCO_3_, DCM, DI water, methanol, and acetonitrile and then dried in a vacuum oven at 60 °C to give a white solid (yield: 80 wt%). DDSQ-Cl: FTIR (KBr, cm^−1^): 1275 (Si-CH_3_), 1135 (Si-O-Si). ^1^H NMR (500 MHz, CDCl_3_, δ, ppm): 7.53–7.44 (aromatic protons), 2.91 (S, 4 H, CH_2_Cl). ^13^C NMR (125 MHz, CDCl_3_, δ, ppm): 134.85, 130.89, 128.86 (aromatic carbons), and 28.65.

### 2.3. Synthesis of p-Xylylene Diazide (DB-N_3_) and Double-Decker Silsesquioxane-N_3_ (DDSQ-N_3_)

A mixture of *p*-dichloro-xylene (3.675 g, 0.021 mol) or DDSQ-Cl (2.625 g, 0.021 mol), NaN_3_ (0.29 g, 0.044 mol), and dry THF (35 mL) was placed in a flask to react under a nitrogen atmosphere and then heated under reflux at 80 °C for 48 h. The solution was filtered to remove any precipitation, and a rotary evaporator was used to remove THF; the product residue was purified by extraction with DCM. The organic layer was separated and added to some DCM (20 mL), and dried with anhydrous MgSO_4_ to stir for 1 h to obtain a white solid (yield: 81%) where DB-N_3_: FTIR (KBr, cm^−1^): 2108 (N_3_). ^1^H NMR (500 MHz, CDCl_3_, δ, ppm): 7.21, 4.35 (4H, CH_2_N_3_). ^13^C NMR (125 MHz, CDCl_3_, δ, ppm): 138.46, 129.38, 45.0. DDSQ-N_3_: FTIR (KBr, cm^−1^): 2095 (N_3_). ^1^H NMR (500 MHz, CDCl_3_, δ, ppm,): 7,67–7.36 (aromatic protons) 2.90 (4H, CH_2_N_3_). ^13^C NMR (125 MHz, CDCl_3_, δ, ppm): 135.93, 130.82, 128.86 (aromatic carbons), 28.63. ^29^Si NMR (600 MHz, CDCl_3_, δ, ppm,): −21.45, −28.04, −79.08. MALDI-TOF MS (calcd. for [M]^+^): *m*/*z* 1272.

### 2.4. Synthesis of 1,4-bis(prop-2-yn-1-yloxy)benzene (P-B), 4,4′-(propane-2,2-diyl)bis((prop-2-yn-1-yloxy)benzene) (P-BPA), and bis(4-(prop-2-yn-1-yloxy)phenyl)methanone (P-CO)

A mixture was formed from hydroquinone (5 g, 0.045 mol) or 4,4′-(propane-2,2-diyl)diphenol (5 g, 0.022 mol) or bis(4-hydroxyphenyl)methanone (5 g, 0.023 mol), and K_2_CO_3_ (8.82 g, 0.064 mol) in acetone (100 mL), and this mixture was stirred for 1 h under nitrogen atmosphere. Then, propargyl bromide (8.602 mL, 0.053 mol) was added and the mixture was heated under reflux at 80 °C for 48 h. The reacting mixture was cooled to room temperature and filtered. After removing the solvent by a rotary evaporator, the sample was purified with silica gel column chromatography (hexane/ethyl acetate = 8/2) to obtain a cream-colored solid product where P-B [Figure S1]: FTIR (KBr, cm^−1^): 3267 (≡C-H), 3105 (C=C-H), 2131 (C≡C). ^1^H NMR (500 MHz, CDCl_3_, δ, ppm): 7.25 (s, 4H), 4.64 (d, J = 2.4 Hz), 2.52 (s, 2H, ≡C-H). ^13^C NMR (125 MHz, CDCl_3_, δ, ppm): 153.09, 116.22, 78.87, 75.04. HR-FD-MS: *m*/*z*: 186 (Figure S2). P-BPA [Figure S3]: FTIR (KBr, cm^−1^): 3267 (≡C-H), 3040 (C=C-H), 2124 (C≡C), 1596 and 1523 (C=C). ^1^H NMR (500 MHz, CDCl_3_, δ, ppm): 7.15 (d, 4H, J = 8.7 Hz), 6.89 (d, 4H, J = 8.7 Hz), 4.67 (d, 4H, O-CH_2_), 2.53 (s, 2H, (≡C-H), 1.62 (s, 6H, CH_3_). ^13^C NMR (125 MHz, CDCl_3_, δ, ppm): 156.66, 144.49, 128.18, 114.54, 79.34, 75.30, 56.36, 41.78, and 31.71 ppm. HR-FD-MS: *m*/*z*: 304.1 (Figure S4). P-CO [Figure S5]: FTIR (KBr, cm^−1^): 3296 (≡C-H), 3073 (C=C-H), 2124 (C≡C), 1644 (C=O), 1596 (C=C). ^1^H NMR (500 MHz, CDCl_3_, δ, ppm): 7.83 (m, 4H), 7.27 (m, 4H), 4.78 (s, 4H, O-CH_2_), 2.54 (s, 2H, ≡C-H). ^13^C NMR (125 MHz, CDCl_3_, δ, ppm): 195.01, 161.23, 132.74, 114.54, 78.14, 76.24, 56.36. HR-MS: *m*/*z*: 291.2 (Figure S6).

### 2.5. Synthesis of 1,2-Diylidenebis(methaneylelidene)bis(3-(prop-2-yn-1-yloxy)phenol) (P-NP)

A mixture was created from 2,4-dihydroxybenzaldehyde (4.0 g, 0.0289 mol), K_2_CO_3_ (8.0 g, 0.0579 mol), and acetonitrile (75 mL) and kept under a nitrogen atmosphere for 1 h. Then, this was injected with propargyl bromide (1.75 mL, 0.0231 mol) to heat under reflux at 80 °C for 24 h. After refluxing overnight under stirring, the reaction mixture was allowed to cool to room temperature. After filtration and removal of all the solvents, the residues were dissolved in DCM and successively washed with water. The organic phase was collected and dried over anhydrous MgSO_4_. After filtration and concentration on a rotary evaporator, the crude product was subjected to further purification by silica gel column chromatography using (hexane/EA = 3/1) as a product. Then, a mixture of 2-hydroxy-4-(prop-2-yn-1-yloxy)benzaldehyde (0.27 g, 0.0015 mol) and hydrazine hydrate solution (0.038 mL, 0.0007 mol) was dissolved in ethanol (30 mL) and the solution mixture was stirred under room temperature for 12 h. After filtration, removal of the solvents, and drying in a vacuum oven, a yellow solid was obtained. P-NP [T_m_: 209 °C by DSC]. FTIR (KBr, cm^−1^): 3279 (≡C-H), 2119 (C≡C). ^1^H NMR (500 MHz, CDCl_3_, δ, ppm): 8.59 (s, 2H, CH=N), 7.3–6.60 (m, 6H), 4.72 (s, 4H), 2.53 (s, 2H, ^13^C NMR (125 MHz, CDCl_3_, δ, ppm): 163.85, 162.43, 134.16, 113.12, 108.30, 102.79, 78.87, 74.56, 55.89. HR-FD-MS: *m*/*z*: 349.2 (Figure S7).

### 2.6. Synthesis of 1,2-bis(4-Hydroxyphenyl)-1,2-diphenylethene (TPE-2OH) and Propargylated-TPE (P-TPE)

TPE-2OH was synthesized according to the McMurry reaction. Here, 4-Hydroxybenzophenone (3 g, 0.015 mol) and Zn dust (5.19 g, 0.08 mol) were added to anhydrous THF (125 mL) under a nitrogen atmosphere at 0 °C for 2 h. After the temperature cooled down to 0 °C, the TiCl_4_ (4.2 mL, 0.038 mol) was added to the mixture immediately under a nitrogen atmosphere and heated under reflux at 70 °C for 24 h. The reaction was cooled to room temperature and anhyd. K_2_CO_3_ (10 g, 0.072 mol) and H_2_O (50 mL) were used to quench the reaction. The cooled mixture was filtered to remove any catalyst. Following this, the THF was removed from the reaction mixture by a rotary evaporator. The organic layer was separated, and the aqueous suspension was extracted with EA (3 × 50 mL). The organic phase was dried with anhydrous MgSO_4_ to afford a white powder. FTIR (KBr, cm^−1^): 3146 (OH), 1596 (C=C). ^1^H NMR (500 MHz, CDCl_3_, δ, ppm): 7.05 (m, 10H), 6.86 (m, 4H), 6.54 (m, 4H). ^13^C NMR (125 MHz, CDCl_3_, δ, ppm): 161.02, 155.28, 154.37, 138.76, 133.56, 130.14, 126.21, 115.78. HR-MS: *m*/*z*: 364.2 (Figure S8). Then, TPE-2OH (3.78 g, 0.010 mol), acetone (50 mL), and K_2_CO_3_ (10.96 g, 0.08 mol)) were stirred for 1 h. Propargyl bromide (2.77 mL, 0.037 mol) was added under an N_2_ atmosphere and then heated under reflux at 60 °C overnight. The reacting mixture was cooled to room temperature and filtered. After the removal of the solvent by a rotary evaporator, it was purified with silica gel column chromatography (hexane/ethyl acetate = 3/1) to obtain a dark brown sticky liquid product. P-TPE [T_m_: 168 °C by DSC]. FTIR (KBr, cm^−1^): 3292 (≡C-H), 2124 (C≡C). ^1^H NMR (500 MHz, CDCl_3_, δ, ppm): 7.2–7.07 (m, 10H), 6.94 (m, 4H), 6.69 (m, 4H), 4.62 (m, 4H), 3.47 (m, 2H, ≡C-H). ^13^C NMR (125 MHz, CDCl_3_, δ, ppm): 156.73, 144.69, 140.21, 137.85, 133.37, 127.98, 114.17, 78.81, 75.58, 60.32. HR-FD-MS: m/z: 440.1 (Figure S9). The melting temperatures of P-B, P-BPA, P-CO, P-NP, and P-TPE were 50.3, 81.6, 91.5, 209, and 168 °C, respectively [Figure S10].

### 2.7. Synthesis of DDSQ-Based Main Chain Type of Copolymer through Click Reaction

A mixture of DDSQ-N_3_ (0.309 g, 0.0002 mol) and CuBr (0.029 g, 0.08 mol) was stirred under a nitrogen atmosphere. After that, DMF (20 mL) was injected into the P-B (0.446 g, 0.0024 mol), P-BPA (0.730 g, 0.0024 mol), P-CO (0.696 g, 0.0024 mol), P-NP (0.836 g, 0.0024 mol), and P-TPE (1.057 g, 0.0024 mol). After using the freeze–thaw cycle method three times, PMDETA (0.0417 mL, 0.08 mol) was injected into the reaction. The mixture reacted under a nitrogen atmosphere and was heated under reflux at 70 °C for 48 h. Then, the solvent of DMF was removed by the reduced pressure distillation system. After removing DMF, the obtained copolymer in the THF solution was passed through an alumina column to remove the CuBr catalyst, and finally, the THF was removed by a rotary evaporator to obtain the desired product.

## 3. Results

### 3.1. Synthesis of DB-N_3_ and DDSQ-N_3_

**Figure 1:**
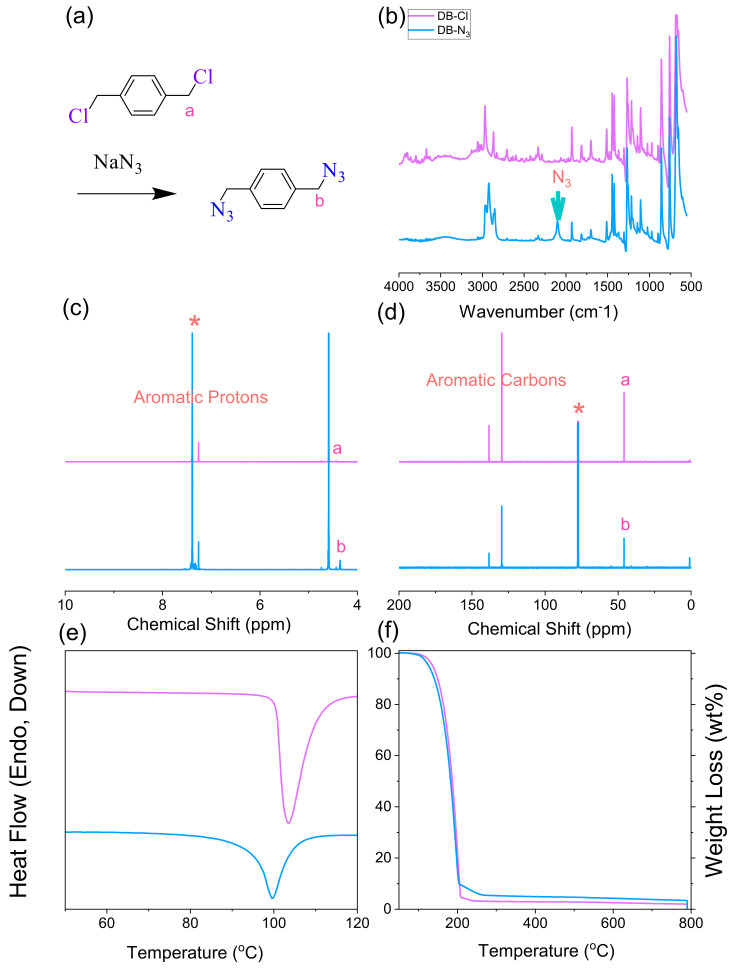
(**a**) Synthesis of DB-N_3_ from DB-Cl, (**b**) FTIR, (**c**) ^1^H-NMR, (**d**) ^13^C-NMR, (**e**) DSC, and (**f**) TGA analyses of DB-Cl and DB-N_3_. * is the CDCl_3_ peak.

**Figure 2:**
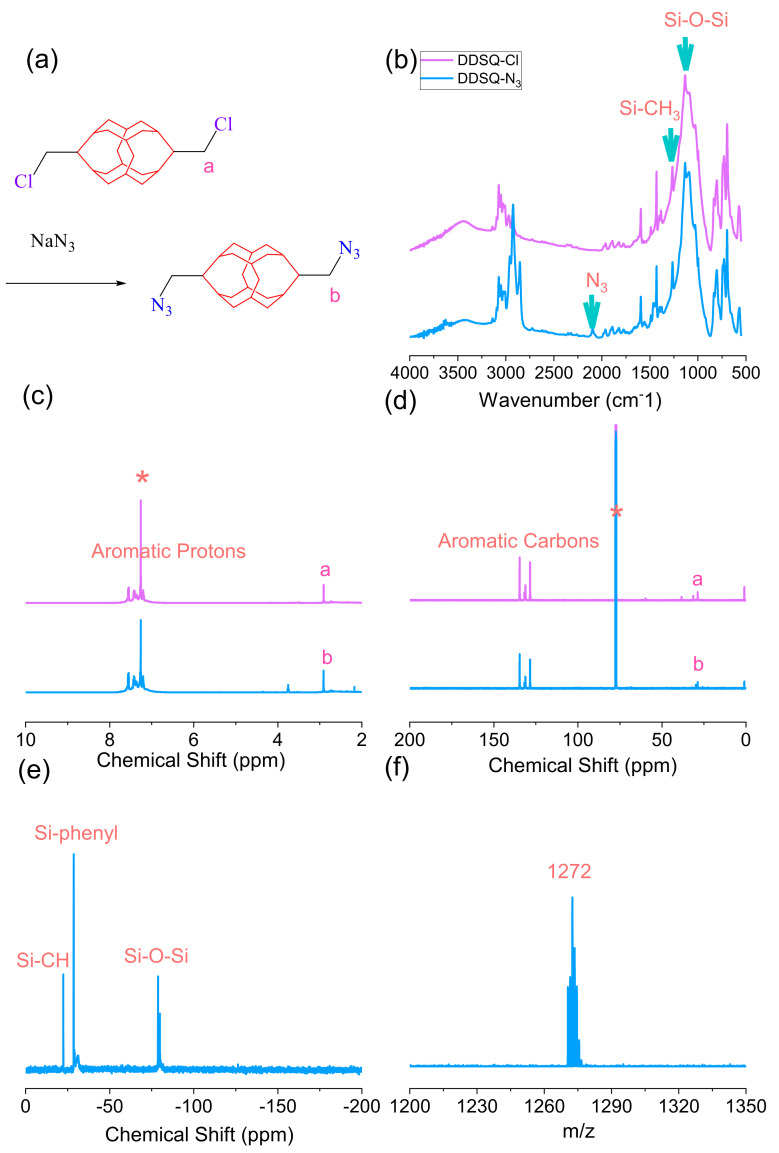
(**a**) Synthesis of DDSQ-N_3_ from DDSQ-Cl, (**b**) FTIR, (**c**) ^1^H-NMR, and (**d**) ^13^C-NMR analyses of DDSQ-Cl and DDSQ-N_3_. (**e**) ^29^Si-NMR solid-state and (**f**) MALDI-TOF profiles of DDSQ-N_3_. * is the CDCl_3_ peak.

The synthesis scheme of DB-N_3_ and DDSQ-N_3_ is shown in Figure 1a and Figure 2a; the absorption peak at 2101 cm^−1^ for DB-N_3_ and 2095 cm^−1^ for DDSQ-N_3_ based on FTIR analysis is shown in Figure 1b and Figure 2b, implying the presence of the azide unit. In addition, the DDSQ-N_3_ also displays the absorption peaks at 1095 cm^−1^ and 1263 cm^−1^ for Si-O-Si and Si-CH_3_ units in Figure 2b. The complete substitution of DB-Cl by the azide unit was also confirmed by ^1^H and ^13^C NMR spectra in Figure 1c,d; the benzyl CH_2_ connected to the Cl atom shifted to a higher field from 4.58 to 4.35 ppm based on ^1^H NMR (Figure 1c) and 45.3 to 45.0 ppm based on ^13^C NMR (Figure 1d) for DB-N_3_. DSC and TGA analyses in Figure 1e,f also indicate that the substitution changed the thermal properties of the azide unit for the DB-N_3_ monomer. Furthermore, Figure 2c,d display ^1^H and ^13^C NMR spectra to confirm the complete substitution of DDSQ-Cl by the azide unit; the DDSQ-CH_2_ connected to the Cl atom was slightly shifted to a higher field from 2.91 to 2.90 ppm based on ^1^H NMR (Figure 2c) and 28.65 to 28.63 ppm based on ^13^C NMR (Figure 2d). The remaining DDSQ units were confirmed using the ^29^Si NMR spectrum as shown in Figure 2e, where the peaks were observed near −21.45, −28.04, and −79.08 ppm, respectively attributed to the Si-CH_2_, Si-Phenyl, and Si-O-Si units in DDSQ-N_3_. Most importantly, the matrix-assisted laser desorption/ionization-time of flight (MALDI-TOF) mass spectra of the DDSQ-N_3_ at 1272 g mol^−1^ for [DDSQ-N_3_ + Na]^+^ was observed, as shown in Figure 2f, and a good correlation existed between the experimental and calculated molecular mass, indicating the well-defined DDSQ structure. Taking into account all data from the FITR, NMR, and MALDI-TOF mass spectra analyses, we could confirm the successful synthesis of DB-N_3_ and DDSQ-N_3_ compounds.

### 3.2. Synthesis of P-B, P-BPA, and P-CO Monomers

Scheme S1 summarizes the synthesis of P-B, P-BPA, and P-CO monomers from hydroquinone, 4,4′-(propane-2,2-diyl)diphenol, and bis(4-hydroxyphenyl)methanone with propargyl bromide at 80 °C under N_2_ atmosphere for 48 h. Figures S1–S3 show their corresponding FTIR, ^1^H, and ^13^C NMR spectra recorded at room temperature. The C≡C-H and C≡C stretching vibration at 3270 and 2130 cm^−1^ for the P-B monomer, 3278 and 2115 cm^−1^ for the P-BPA monomer, and 3270 and 2125 cm^−1^ for the P-CO monomer and the other characteristic absorption peaks including C=C and C=O units are summarized in Figures S1b, S2b, and S3b, respectively, based on FTIR analyses. ^1^H NMR spectra display the peaks of O-CH_2_ and C≡C-H at 4.65, and 2.50 ppm for the P-B monomer, 4.65 and 2.51 ppm for the P-BPA monomer, and 4.78 and 2.56 ppm for the P-CO monomer, and the other characteristic peaks, including aromatic protons and CH_3_ protons, are also summarized in Figures S1c, S2c, and S3c. Furthermore, their corresponding ^13^C NMR in Figures S1d, S2d, and S3d exhibited peaks of O-CH_2_ and C≡C at 56.53, 79.42, and 75.46 ppm for P-B monomer, 55.90, 79.21, and 75.60 ppm for P-BPA monomer, and 56.10, 77.78, and 76.40 ppm for P-CO monomer. The other carbon peaks including C=O, aromatic carbon, and CH_3_ were also summarized. All these results are able to confirm the successful synthesis of P-B, P-BPA, and P-CO monomers in this study.

### 3.3. Synthesis of P-NP Monomer

**Figure 3:**
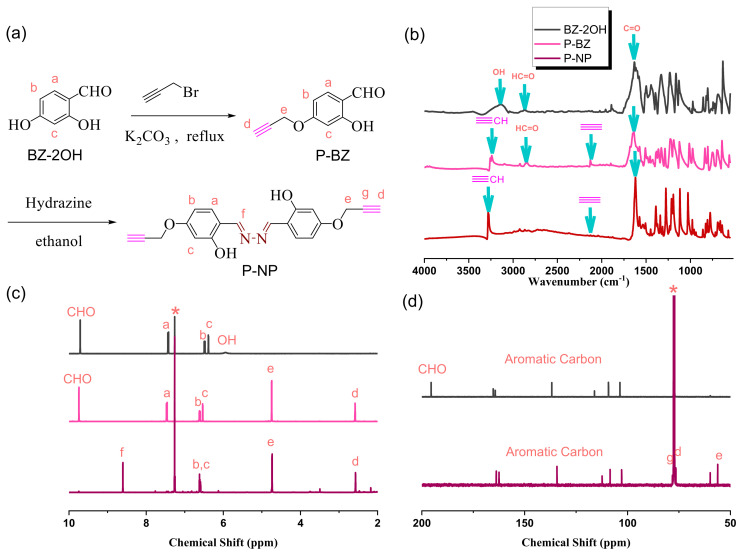
(**a**) Synthesis of P-NP from BZ-2OH and P-BZ, (**b**) FTIR, (**c**) ^1^H-NMR analyses of BZ-2OH, P-BZ, and P-NP and (**d**) ^13^C-NMR profiles of BZ-2OH and P-BZ. * is the CDCl_3_ peak.

The synthesis of P-NP is summarized in Figure 3a. Here, 2,4-dihydroxybenzaldehyde (BZ-2OH) is used as a starting material and then reacted with propargyl bromide to form 2-hydroxy-4-(prop-2-yn-1-yloxy)benzaldehyde (P-BZ). The P-BZ reacted with hydrazine hydrate solution to form a P-NP monomer. Figure 3b shows the corresponding FTIR spectra of 2,4-dihydroxybenzaldehyde, 2-hydroxy-4-(prop-2-yn-1-yloxy)benzaldehyde, and P-NP monomer where the absorption peaks are seen at 3136, 2871, and 1634 cm^−1^ due to the OH, CHO, and C=O units for the 2,4-dihydroxybenzaldehyde compound. The OH absorption was decreased and formed the C≡C-H and C≡C units at 3243 and 2127 cm^−1^ for 2-hydroxy-4-(prop-2-yn-1-yloxy)benzaldehyde compound. After the Schiff-base reaction with hydrazine, the CHO absorption intensity at 2871 cm^−1^ was decreased, and the C=N absorption intensity at 1615 cm^−1^ became obvious for the P-NP monomer. Figure 3c displays the ^1^H NMR spectra of the P-NP monomer at 9.70 and 5.94 ppm corresponding to CHO and OH units, and the other aromatic protons ranging from 7.43 to 6.39 ppm. The OH units disappeared and formed O-CH_2,_ and C≡C-H units at 4.74 and 2.59 ppm, and the other protons, such as CHO and aromatic protons, remained for 2-hydroxy-4-(prop-2-yn-1-yloxy)benzaldehyde compound. After the Schiff-base reaction with hydrazine, the CHO proton at 9.70 ppm disappeared, and the CH=N (peak *f*) was found at 8.61 ppm to form a P-NP monomer. Similarly, ^13^C NMR spectra of 2,4-dihydroxybenzaldehyde and the P-NP monomer are shown in Figure 3d. The CHO carbon was observed at 195.22 ppm, and the aromatic carbon ranging from 165.10 to 103.68 ppm; however, the CHO carbon disappeared and the O-CH_2_ and C≡C carbon were seen at 56.22, 78.10, and 76.40 ppm for the P-NP monomer, indicating the successful synthesis of the P-NP monomer in this study.

### 3.4. Synthesis of P-TPE Monomer

**Figure 4:**
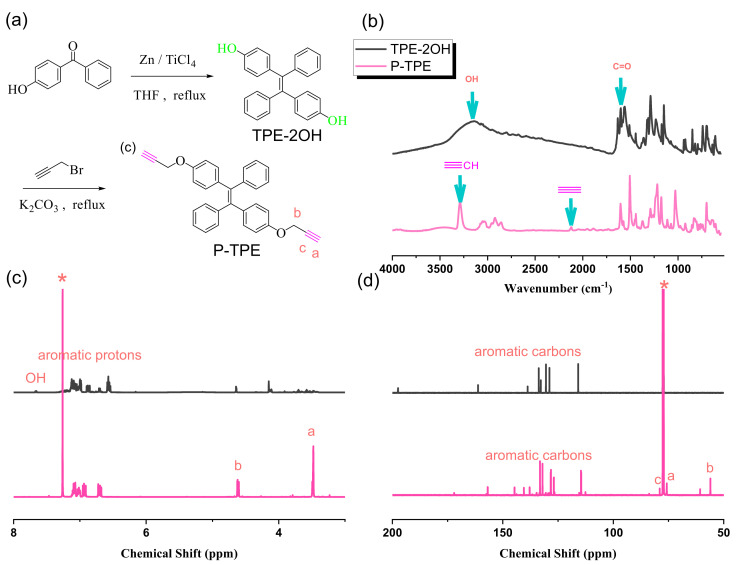
(**a**) Synthesis of TPE-2OH and P-TPE, (**b**) FTIR, (**c**) ^1^H-NMR, and (**d**) ^13^C-NMR analyses of TPE-2OH and P-TPE. * is the CDCl_3_ peak.

The synthesis of P-TPE is summarized in Figure 4a. Here, 4-dydroxybenzophenone is reacted with Zn and TiCl_4_ to form TPE-2OH based on the McMurry reaction. Similarly, the TPE-2OH reacted with propargyl bromide to form a P-TPE monomer. The OH and C=O stretching vibrations were seen at 3142 and 1637 cm^−1^ for the 4-dydroxybenzophenone compound, and both peaks disappeared and formed the ≡C-H and C≡C stretching vibration at 3289 and 2118 cm^−1^ for the P-TPE monomer based on FTIR analyses in Figure 4b. Figure 4c shows the ^1^H NMR spectra of 4-dydroxybenzophenone at 7.65 ppm, and the other aromatic protons ranging from 7.13 to 6.56 ppm. The OH unit disappeared and formed the O-CH_2_ and C≡C-H units at 4.61 and 3.47 ppm of P-TPE. In addition, ^13^C NMR spectra of 4-dydroxybenzophenone and the P-TPE monomer are shown in Figure 4d. The C=O carbon at 197.29 ppm was observed and the aromatic carbon ranged from 162.27 to 115.88 ppm; the C=O carbon disappeared and the O-CH_2_ and C≡C carbon were observed at 56.09, 78.91, and 75.77 ppm for the P-TPE monomer, also indicating the successful synthesis of the P-TPE monomer in this study.

### 3.5. Synthesis of TPE-DB-Based Main Chain Type of Copolymer through Click Reaction

**Figure 5:**
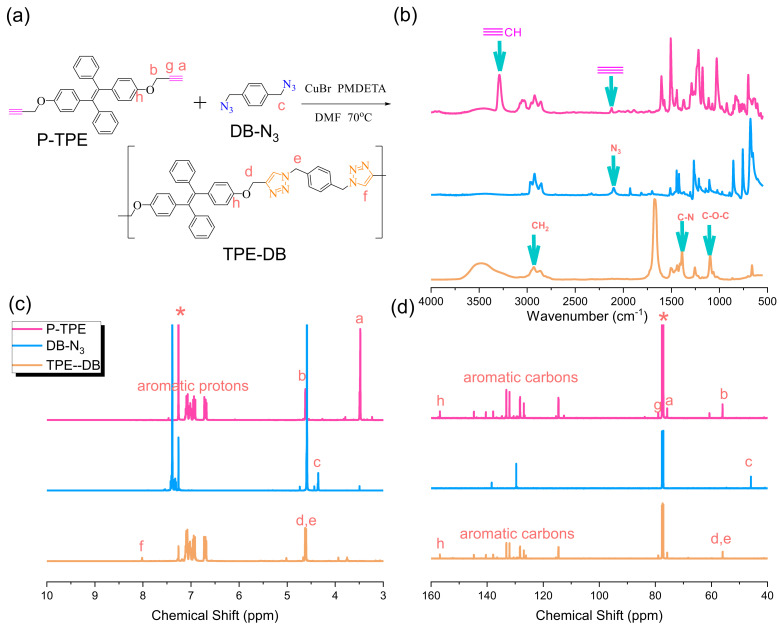
(**a**) Synthesis of TPE-DB from P-TPE and DP-N_3_, (**b**) FTIR, (**c**) ^1^H-NMR, and (**d**) ^13^C-NMR analyses of P-TPE and DP-N_3_ and TPE-DB. * is the CDCl_3_ peak.

In this study, we first synthesized the TPE-DB copolymer through click reaction from P-TPE and DB-N_3_ as the model reaction as shown in Figure 5a under an N_2_ atmosphere at 70 °C for 48 h. The chemical structure of the P-TPE and DB-N_3_ monomers has already been discussed in detail above, as shown in Figure 1 and Figure 4, based on FTIR, ^1^H, and ^13^C NMR analyses. The total disappearance of the characteristic acetylene unit at 3142 cm^−1^ and 2118 cm^−1^ of P-TPE, and the azide unit of the DB-N_3_ unit at 2101 cm^−1^ based on FTIR analyses in Figure 5b, suggest that both acetylene and azide units participated in the click reaction. In addition, Figure 5c shows their ^1^H NMR spectra; we could also observe the appearance of a new signal at 8.04 ppm, corresponding to the proton of the triazole unit from the click reaction. Furthermore, the CH_2_ unit from DB-N_3_ was significantly down-field shifted from 4.35 to 4.61 ppm. The disappearance of the C≡C-H unit at 3.47 ppm from P-TPE and the corresponding ^13^C NMR spectra are also summarized in Figure 5d; the CH_2_ unit from DB-N_3_ was significantly down-field shifted from 45.91 to 56.09 ppm, and there was a decrease in acetylene units, also indicating that the synthesis of the TPE-DB copolymer was successful.

### 3.6. Synthesis of TPE-DDSQ-Based Main Chain Type of Copolymer through Click Reaction

**Figure 6:**
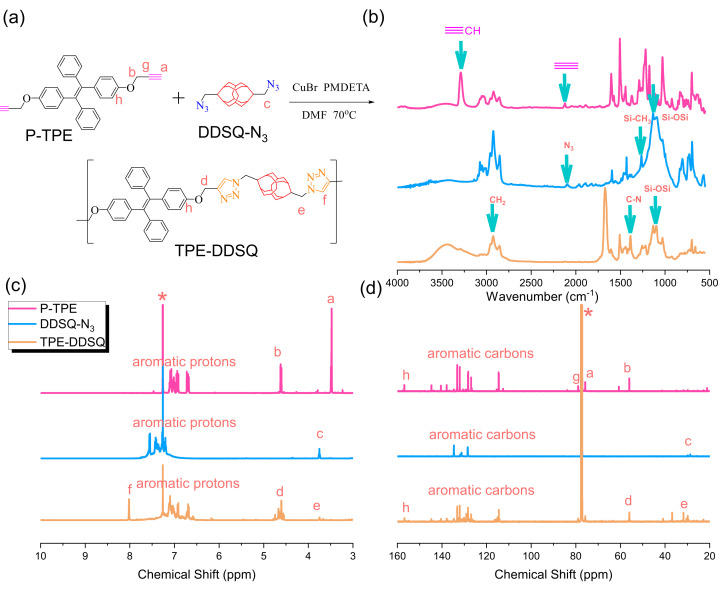
(**a**) Synthesis of TPE-DDSQ from P-TPE and DDSQ-N_3_, (**b**) FTIR, (**c**) ^1^H-NMR, and (**d**) ^13^C-NMR analyses of P-TPE and DDSQ-N_3_ and TPE-DDSQ. * is the CDCl_3_ peak.

Similar to the TPE-DB copolymer, the synthesis of the TPE-DDSQ main chain type of copolymer with an inorganic DDSQ cage structure from P-TPE with DDSQ-N_3_ by using click reaction is displayed in Figure 6a. The total disappearance of the characteristic acetylene unit at 3142 cm^−1^ and 2118 cm^−1^ of P-TPE and the azide unit of the DDSQ-N_3_ unit at 2095 cm^−1^ based on FTIR analyses is shown in Figure 6b. The remaining Si-O-Si stretching absorption at 1095 cm^−1^ of the TPE-DDSQ copolymer suggests the successful synthesis of the TPE-DDSQ copolymer, and both acetylene and azide units participated in the click reaction. Furthermore, the appearance of a new signal at 8.01 ppm corresponds to the proton of the triazole unit from the click reaction based on ^1^H NMR analysis in Figure 6c. The CH_2_ unit from DDSQ-N_3_ was slightly down-field shifted from 28.70 to 31.81 ppm, and the decrease in acetylene units based on ^13^C NMR analyses in Figure 6d also implies that the synthesis of the TPE-DDSQ copolymer was successful.

### 3.7. Synthesis of Other DDSQ-Based Main Chain Types of Copolymer through Click Reaction

**Figure 7:**
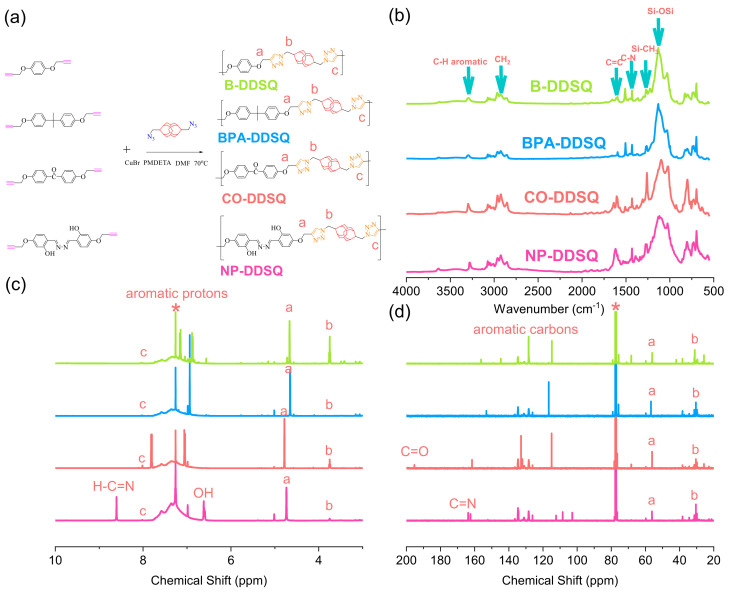
(**a**) Synthesis of B-DDSQ, BPA-DDSQ, CO-DDSQ, and NP-DDSQ. (**b**) FTIR, (**c**) ^1^H-NMR, and (**d**) ^13^C-NMR analyses of B-DDSQ, BPA-DDSQ, CO-DDSQ, and NP-DDSQ. * is the CDCl_3_ peak.

The synthesis of other DDSQ-based copolymers, such as B-DDSQ, BPA-DDSQ, CO-DDSQ, and NP-DDSQ organic/inorganic hybrids is summarized in Figure 7a. The disappearance of the characteristic acetylene unit at 2115–2130 cm^−1^ of propargyl-functionalized monomers and the azide unit of the DDSQ-N_3_ unit at 2095 cm^−1^ based on FTIR analyses is shown in Figure 7b. The remaining Si-O-Si and Si-CH_3_ stretching absorption at 1095 cm^−1^ and 1263 cm^−1^ of the other four DDSQ-based copolymers also suggested that both acetylene and azide units participated in the click reaction. In addition, the small signal at ca. 8.01 ppm is due to the protons of triazole units of these DDSQ-based copolymers from the click reaction based on ^1^H NMR analysis in Figure 7c. Both CH_2_ (*a* for O-CH_2_ and *b* for N-CH_2_) units were located at ca. 4.77–4.65 ppm and 3.75–3.74 ppm, respectively, and other aromatic protons also ranged from 7.85 to 6.81 ppm. Furthermore, the NP-DDSQ copolymer shows further CH=N and OH protons at 8.61 and 6.61 ppm, implying that the synthesis of the NP-DDSQ copolymer was successful. The corresponding ^13^C NMR spectra are also summarized in Figure 7d. The C=O unit was observed at 195.44 ppm for the CO-DDSQ copolymer, and the C=N unit was located at 164.17 ppm for NP-POSS. Both CH_2_ (*a* for O-CH_2_ and *b* for N-CH_2_) units were located at ca. 56.70–56.06 ppm and 31.03–30.29 ppm, respectively, and other aromatic carbon signals are also observed in Figure 7d. All results from FTIR and NMR analyses indicate that all DDSQ-based copolymers were successfully synthesized in this study. Table 1 and Table S1 summarize the molecular weights, PDI [by GPC and MALDI-TOF analyses, Figures S11–S15], and thermal properties of all DDSQ copolymers synthesized in this study.

### 3.8. Thermal Property and Morphology Analyses of DDSQ-Based Copolymers

**Figure 8:**
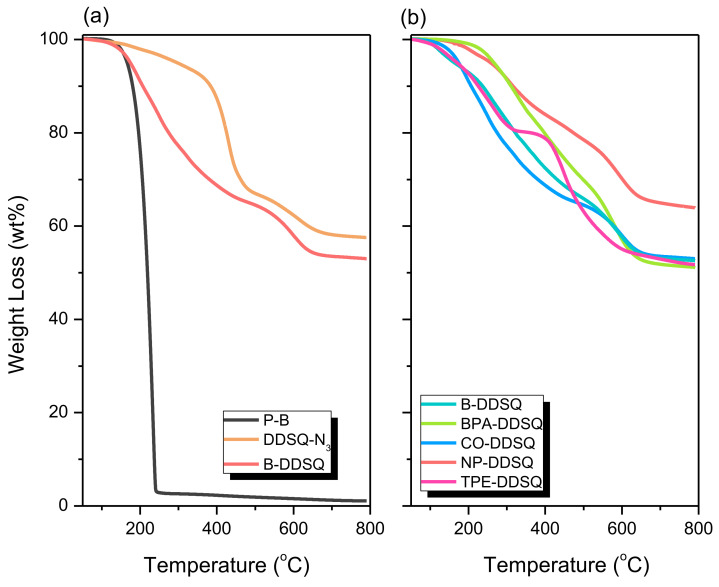
TGA analyses of P-B, DDSQ-N_3_, B-DDSQ (**a**) and (**b**) B-DDSQ, BPA-DDSQ, CO-DDSQ, NP-DDSQ, and TPE-DDSQ.

The thermal stability of these DDSQ-based copolymers under an N_2_ atmosphere was measured by TGA analyses, as shown in Figure 8. Figure 8a shows TGA analyses of P-B and DDSQ-N_3_ monomers, and the corresponding B-DDSQ copolymer after the click reaction. The P-B monomer exhibited very low thermal stability with *T*_d10_ = 180 °C, char yield = 1.1 wt%; however, the DDSQ-N_3_ monomer displayed relatively higher thermal stability with *T*_d10_ = 383 °C, char yield = 57.5 wt% since the inorganic DDSQ cage in the DDSQ-N_3_ monomer could improve the thermal resistance behavior. After the click reaction to form a B-DDSQ copolymer, it also exhibited high thermal stability with *T*_d10_ = 206 °C, which is higher than the P-B monomer and the char yield = 53.1 wt%, which is close to the char yield of the DDSQ-N_3_ monomer. The difference in the *T*_d_ value might be due to the effect of creating the hybrid property in the organic P-B monomer. In hybrid materials, the thermal motion is restricted, reducing the organic material’s decomposition pathways. The inorganic DDSQ would provide the additional heat capacity and stabilize the higher thermal decomposition. In addition, the char yield of the B-DDSQ copolymer would be enhanced after the click reaction because the covalent bond of the DDSQ cage with the P-B monomer also restricts the thermal motion of this hybrid material, indicating that the thermal stability of organic materials could improve through the click reaction and the inorganic silsesquioxane [1,3,22]. All TGA analyses of other DDSQ-based copolymers are also summarized in Figure 8b, which all display high char yields of more than 50 wt%, and the NP-POSS displays the highest char yield with 65.1 wt%. This phenomenon may be due to the strong intramolecular hydrogen bonding of the OH–N units of the P-NP monomer compared with other propargyl-functionalized monomers, as expected.

**Figure 9:**
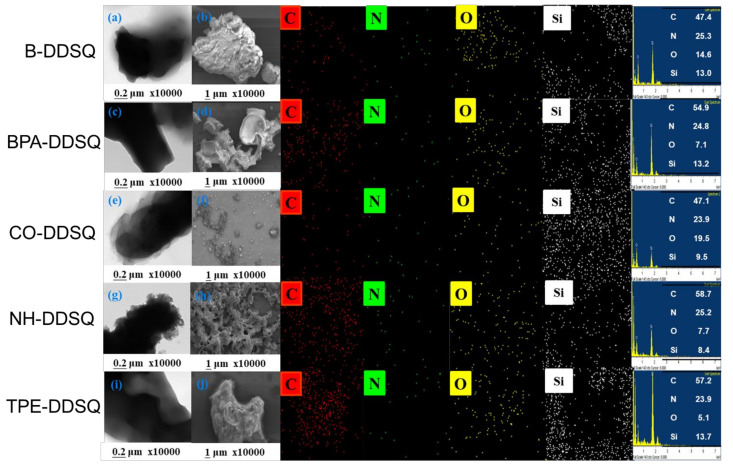
TEM and SEM images of B-DDSQ (**a**,**b**), BPA-DDSQ (**c**,**d**), CO-DDSQ (**e**,**f**), NP-DDSQ (**g**,**h**), and TPE-DDSQ (**i**,**j**).

The inorganic DDSQ cage dispersion into the main chain of copolymers was investigated by TEM and SEM analyses, as shown in Figure 9. No strong aggregation and featureless morphology without phase separation suggested that the inorganic DDSQ cages were dispersed well into the DDSQ-based copolymers due to the covalent bond of the DDSQ cage in these main chain types of DDSQ-based copolymers. Furthermore, the C-, N-, O-, and Si-mapping from SEM images suggest that the DDSQ was uniformly dispersed on the copolymer surfaces. The white points correspond to the DDSQ-rich domain, also confirmed by the TEM image. Each element of composition is also summarized in Figure 9 for all DDSQ-based copolymers. The homogeneous dispersions of inorganic DDSQ cages in the copolymer could decrease the thermal motions and improve the thermal stability, which is consistent with TGA analyses.

### 3.9. Photoluminescence Property of NP-DDSQ and TPE-DDSQ Copolymers

**Figure 10:**
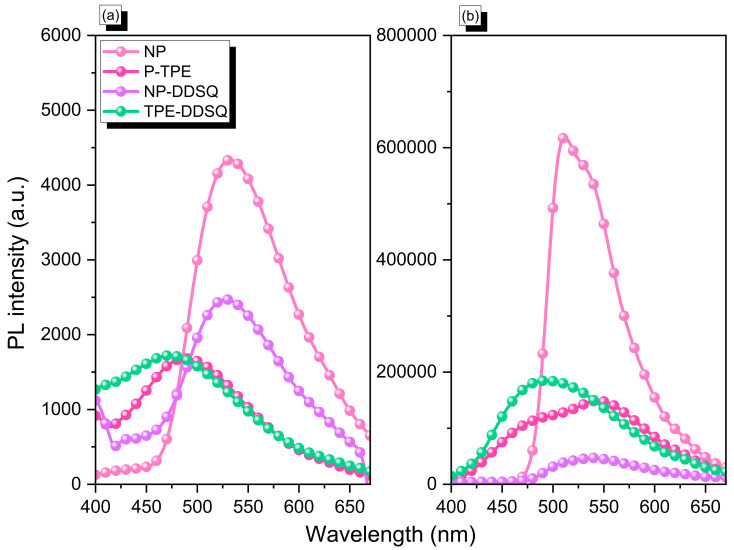
PL spectra of NP, P-TPE, NP-DDSQ, and TPE-DDSQ in THF solution (**a)** and solid state (**b**) [excitation wavelength was 365 nm].

In this study, we choose NP-DDSQ and TPE-DDSQ copolymers to investigate the PL emission properties in solution and solid state since these two units of DP and TPE have photoluminescence properties [48,49,50,51,52,53,54,55,56,57,58]. Firstly, we measured the PL emission of NP, P-TPE, NP-DDSQ, and TPE-DDSQ in THF concentration (10^−6^ M), which exhibited PL peaks centered at 530, 480, 540, and 490 nm for NP, P-TPE, NP-DDSQ, and TPE-DDSQ, respectively [Figure 10a]. In addition, the PL analyses in the solid state [Figure 10b] exhibited emission bands at 510, 541, 540, and 490 nm, respectively, for NP, P-TPE, NP-DDSQ, and TPE-DDSQ. Furthermore, under a UV lamp with wavelength excitation (365 nm), both NP and NP-DDSQ copolymers emitted a green color, and P-TPE and TPE-DDSQ emitted a cyan color, as shown in Figure S16. Interestingly, the NP-DDSQ copolymer shows a strong emission peak at 540 nm due to the intramolecular hydrogen bonding of OH–N and aromatic ring units and excited-state intramolecular proton transfer (ESIPT) character [50,51,52,59]. The fluorescence spectra of two materials, namely NP-DDSQ and TPE-DDSQ copolymers, which were measured at the particular excitation wavelength of 360 nm are shown in Figure S17. It is to be noted that when these materials were prepared at different concentrations in organic solvents such as THF, they showed different emission centers. As a result, we observed that the NP-DDSQ copolymer showed strong emission at around 525 nm for the concentration 10^−5^ M and TPE-DDSQ copolymer showed a strong emission center at around 425 nm for the concentration 10^−5^ M. The fluorescence intensity of both the NP-DDSQ and TPE-DDSQ copolymers increased with increasing water contents as shown in Figure S18. Additionally, to support these phenomena, we also investigated the fluorescence lifetime of the corresponding samples NP-DDSQ, and TPE-DDSQ, and the values were found to be 1.468 ns and 0.885 ns, respectively (Figures S19 and S20).

## 4. Conclusions

This study showed various successfully synthesized DDSQ-based copolymers based on click reaction through DDSQ-N_3_ monomer with different propargyl-functionalized monomers. FTIR, NMR, and GPC analyses were characterized by their chemical structure and molecular weight. The results indicate that the incorporation of the inorganic DDSQ cage of these copolymers could improve their thermal stability properties, such as thermal decomposition temperature and char yield, because of the homogeneity of DDSQ dispersion in the copolymer matrix, and could also affect the optical properties of the NP and TPE units in this work. In addition, both the NP-DDSQ and TPE-DDSQ copolymers could be considered good materials for metal sensing and biomedical applications.

## Figures and Tables

**Table 1:** Summarized molecular weights, PDI, and thermal properties of all synthesized DDSQ copolymers in this study.

Sample	*T*_d10_ (°C)	Char Yield (wt%)	*M*_n_ (g/mol)	PDI	*T*_g_
B-DDSQ	206	53.5	3270	1.30	94
BPA-DDSQ	235	53.0	2930	1.54	65
CO-DDSQ	309	51.6	3600	1.31	67
NP-DDSQ	315	65.1	4860	1.24	89
TPE-DDSQ	383	57.5	4720	1.10	168

## Data Availability

The data presented in this study are available on request from the corresponding author.

## Supplementary Materials

The following supporting information can be downloaded at: https://www.mdpi.com/article/10.3390/polym15020331/s1, Table S1: MALDI TOF results of B-DDSQ, BPA-DDSQ, CO-DDSQ, NP-DDSQ, and TPE-DDSQ copolymers; Scheme S1: Synthesis of (a) P-B, (b) P-BPA, and (c) P-CO monomers; Scheme S2: Synthesis of (a) P-NP and (b) P-TPE monomers; Figure S1: (a) chemical structure, (b) FTIR, (c) 1H NMR, and (d) 13C NMR spectra of P-B; Figure S2: High Resolution-FD-MS spectrum of P-B; Figure S3: (a) chemical structure, (b) FTIR, (c) 1H NMR, and (d) 13C NMR spectra of P-BPA; Figure S4: High Resolution-FD-MS spectrum of P-BPA; Figure S5: (a) chemical structure, (b) FTIR, (c) 1H NMR, and (d) 13C NMR spectra of P-CO; Figure S6: High-Resolution MS spectrum of P-CO; Figure S7: High Resolution-FD-MS spectrum of P-NP; Figure S8: High-Resolution MS spectrum of TPE-2OH; Figure S10: DSC profiles of P-B, P-BPA, P-CO, P-NP, and P-TPE; Figure S11: MALDI TOF profile of B-DDSQ; Figure S12: MALDI TOF profile of BPA-DDSQ; Figure S13: MALDI TOF profile of CO-DDSQ; Figure S14: MALDI TOF profile of NP-DDSQ; Figure S15: MALDI TOF profile of TPE-DDSQ; Figure S16: Photo images of NP, P-TPE, NP-DDSQ, and TPE-DDSQ samples under a UV lamp with an excitation wavelength of 365 nm; Figure S17: PL spectra of NP-DDSQ (a), (b) TPE-DDSQ, and (c) photo images of NP-DDSQ and TPE-DDSQ in THF solution with different concentrations from 10−2 to 10−6 M; Figure S18: PL spectra of (a) NP-DDSQ and (b) TPE-DDSQ copolymers in THF solution (10−5 M) with different water contents; Figure S19: Fluorescence lifetime decay of NP-DDSQ copolymer; Figure S20: Fluorescence lifetime decay of TPE-DDSQ copolymer.
