# Supplementary material for: Construction of Fluorescent Conjugated Polytriazole Containing Double-Decker Silsesquioxane: Click Polymerization and Thermal Stability
Source: Polymers (Basel). 2023 Jan 9;15(2):331. doi: 10.3390/polym15020331 (PMC9863912; doi:10.3390/polym15020331)
Supplement: Supplementary file 1 [file polymers-15-00331-s001.zip › polymers-2116341-supplementary.pdf]

Supporting Information

Construction of Fluorescent Conjugated Polytriazole Containing Double-Decker Silsesquioxane: Click Polymerization and Thermal Stability

Chia-Husan Chiang[1], Mohamed Gamal Mohamed[1,2,*], Wei-Cheng Chen[1], Manivannan Madhu[3], Wei-Lung Tseng[3] and Shiao-Wei Kuo[1,4,*]

[1]Department of Materials and Optoelectronic Science, College of Semiconductor and Advanced Technology Research, Center for Functional Polymers and Supramolecular Materials, National Sun Yat-Sen University, Kaohsiung 804, Taiwan

[2]Chemistry Department, Faculty of Science, Assiut University, Assiut, Egypt

[3]Department of Chemistry, National Sun Yat-sen University, Kaohsiung 804, Taiwan

[4]Department of Medicinal and Applied Chemistry, Kaohsiung Medical University, Kaohsiung 807, Taiwan

Corresponding authors:

E-mail: mgamal.eldin34@gmail.com (M. G. Mohamed), and (kuosw@faculty.nsysu.edu.tw (S. W. Kuo).

Characterization

FTIR spectra were recorded using a Bruker Tensor 27 FTIR spectrophotometer with a resolution of 4 cm^{-1} through the KBr disk method. ^{1}H and ^{13}C Nuclear magnetic resonance (NMR) spectra were recorded using an INOVA 500 instrument with $CDCl_3$

as the solvent and TMS as the external standard; chemical shifts are reported in parts per million (ppm). Dynamic thermal curing kinetics were measured using a TA Q-20 differential scanning calorimeter with ca. 7 mg sample on the DSC sample pan under a N_2 atmosphere (100 mL min^{-1}), with heating from -80 to 350 °C at a heating rate of 10 °C min^{-1}. The thermal stabilities of the samples were examined under a N_2 using a TA Q-50 thermogravimetric analyzer; each cured sample (ca. 5 mg) was placed in a Pt cell and heated at a rate of 20 °C min^{-1} from 40 to 800 °C under a N_2 flow rate of 60 mL min^{-1}. The morphologies of the polymer samples were examined using scanning electron microscopy (SEM; JEOL-6330) and using transmission electron microscopy (TEM; JEOL-2100) instrument operated at an accelerating voltage of 200 kV. Emission spectra of all samples were recorded using Horiba FluoroMax4+ apparatus (integration time: 0.1 s, slit width: 3 nm) with Fluoromax software.

Table S1. MALDI TOF results of B-DDSQ, BPA-DDSQ, CO-DDSQ, NP-DDSQ and TPE-DDSQ copolymers.

Sample name	M_n	M_w	PDI
B-DDSQ	2262	2919	1.288
BPA-DDSQ	2291	2975	1.298
CO-DDSQ	2218	2867	1.292
NP-DDSQ	2219	2844	1.281
TPE-DDSQ	2129	2717	1.276

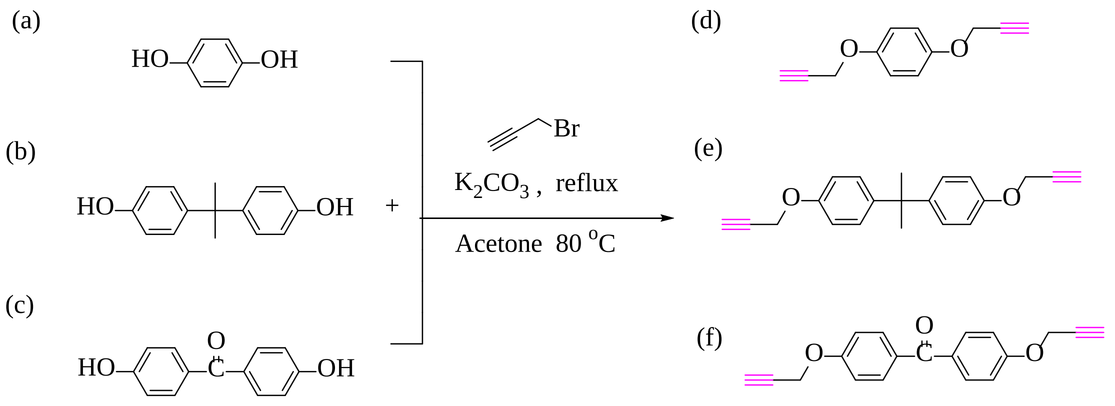

Scheme S1. Synthesis of (a) P-B, (b) P-BPA, and (c) P-CO monomers.

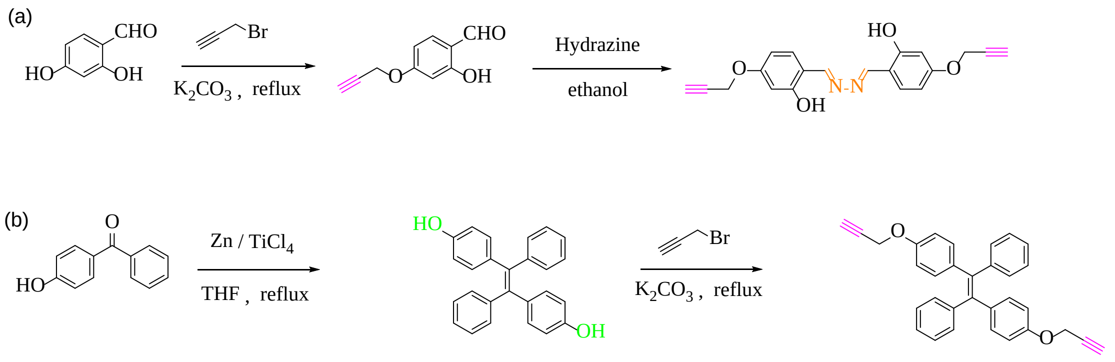

Scheme S2. Synthesis of (a) P-NP, and (b) P-TPE monomers.

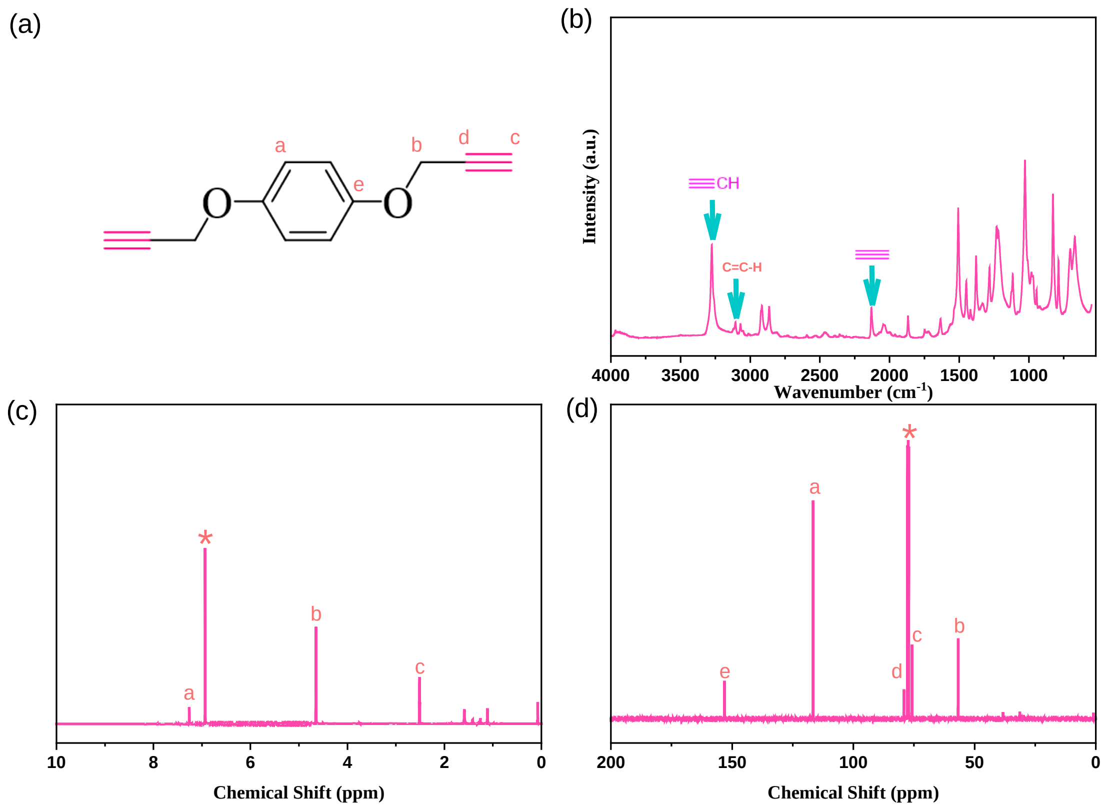

Figure S1. (a) chemical structure, (b) FTIR, (c) ^{1}H NMR, and (d) ^{13}C NMR spectra of P-B. * is the $CDCl_3$ peak.

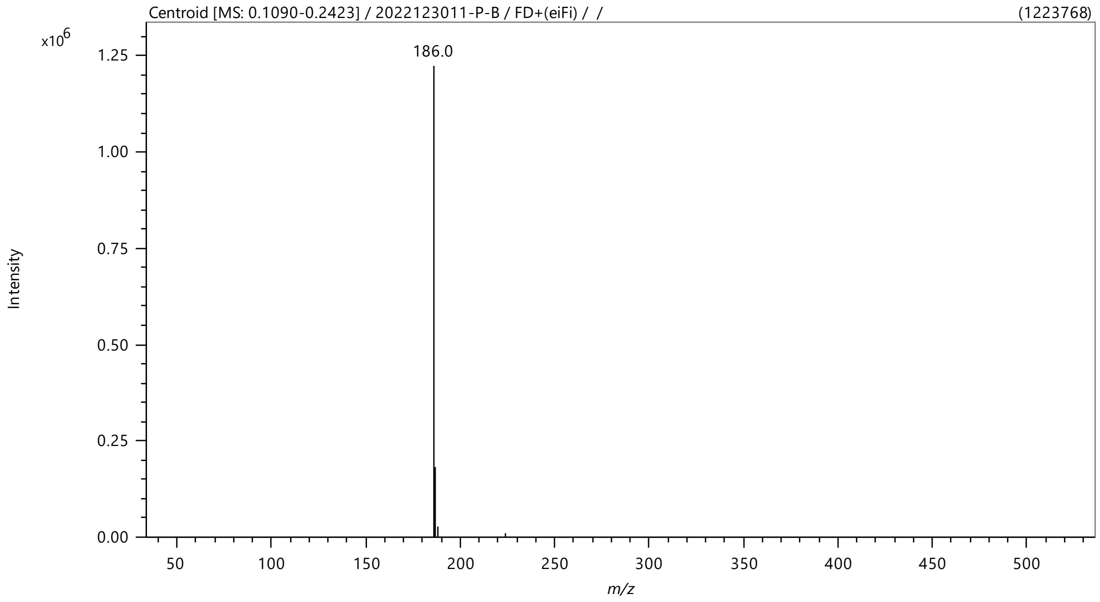

Figure S2. High Resolution-FD-MS spectrum of P-B.

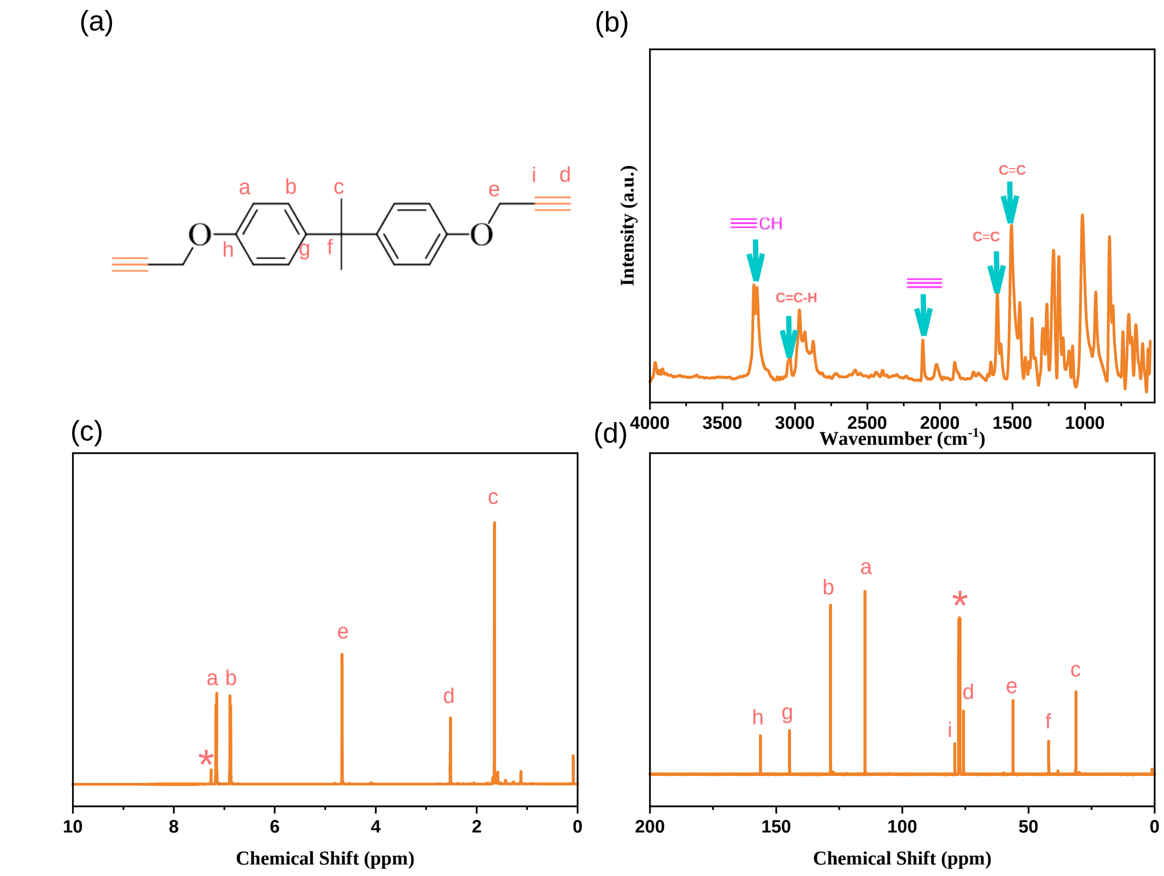

Figure S3. (a) chemical structure, (b) FTIR, (c) ^{1}H NMR, and (d) ^{13}C NMR spectra of P-BPA. * is the $CDCl_3$ peak.

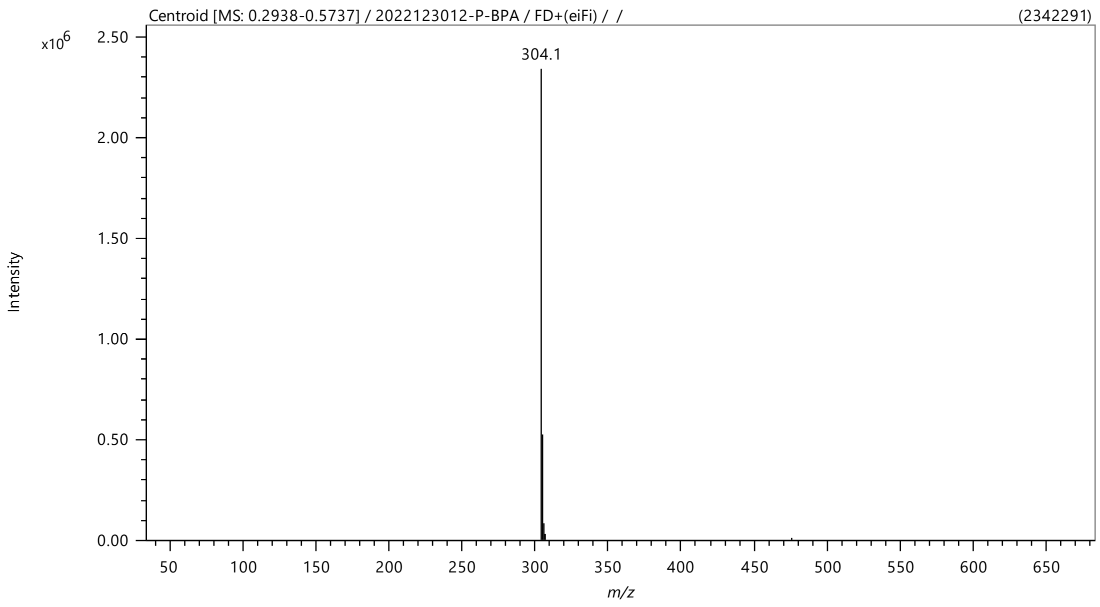

Figure S4. High Resolution-FD-MS spectrum of P-BPA.

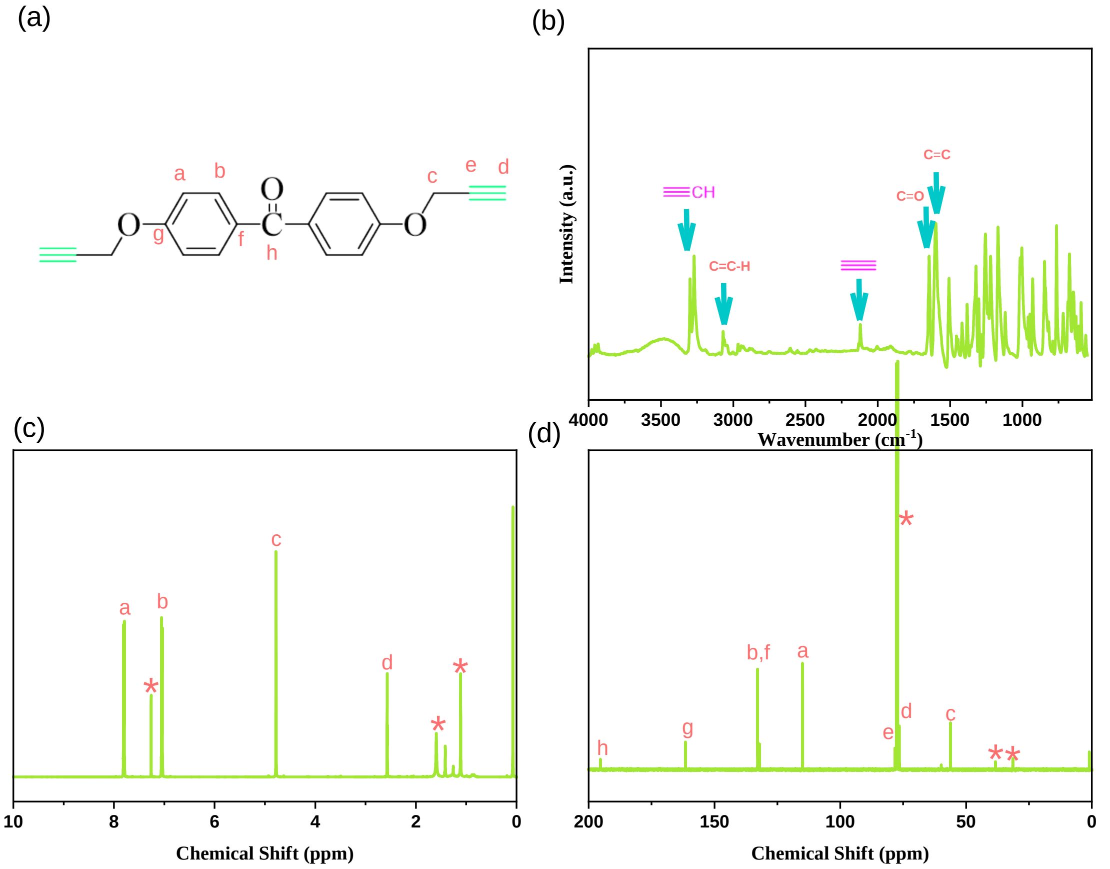

Figure S5. (a) chemical structure, (b) FTIR, (c) ^{1}H NMR, and (d) ^{13}C NMR spectra of P-CO. * is the $CDCl_3$ peak.

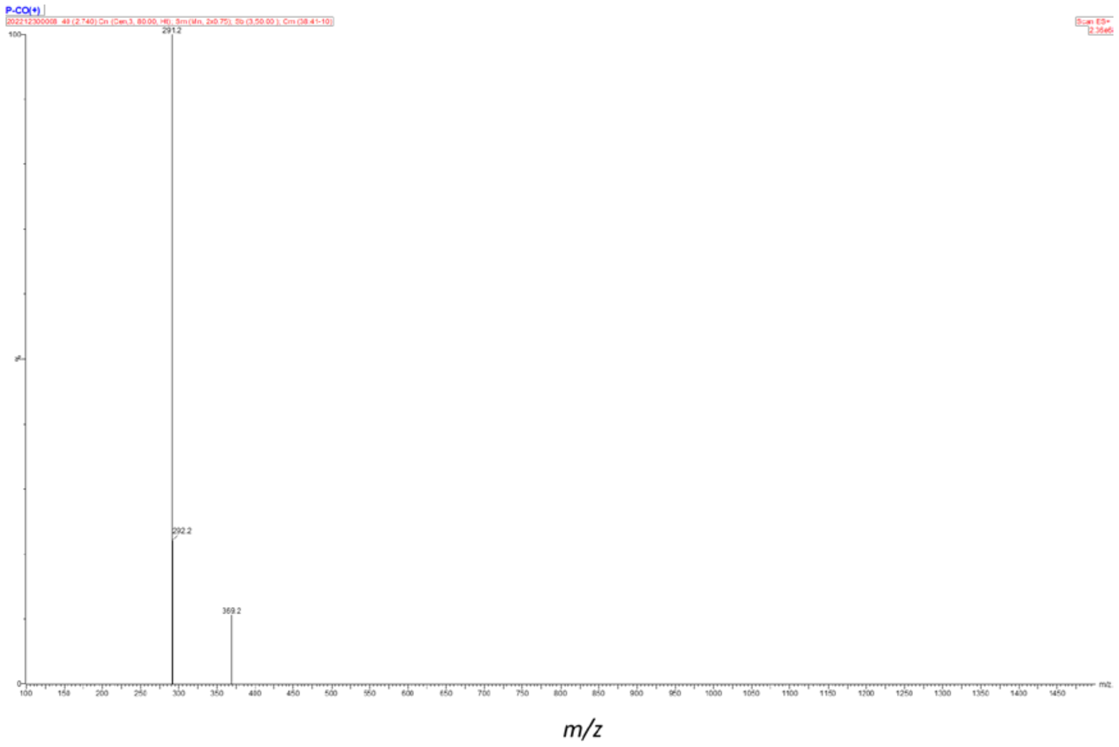

Figure S6. High-Resolution MS spectrum of P-CO.

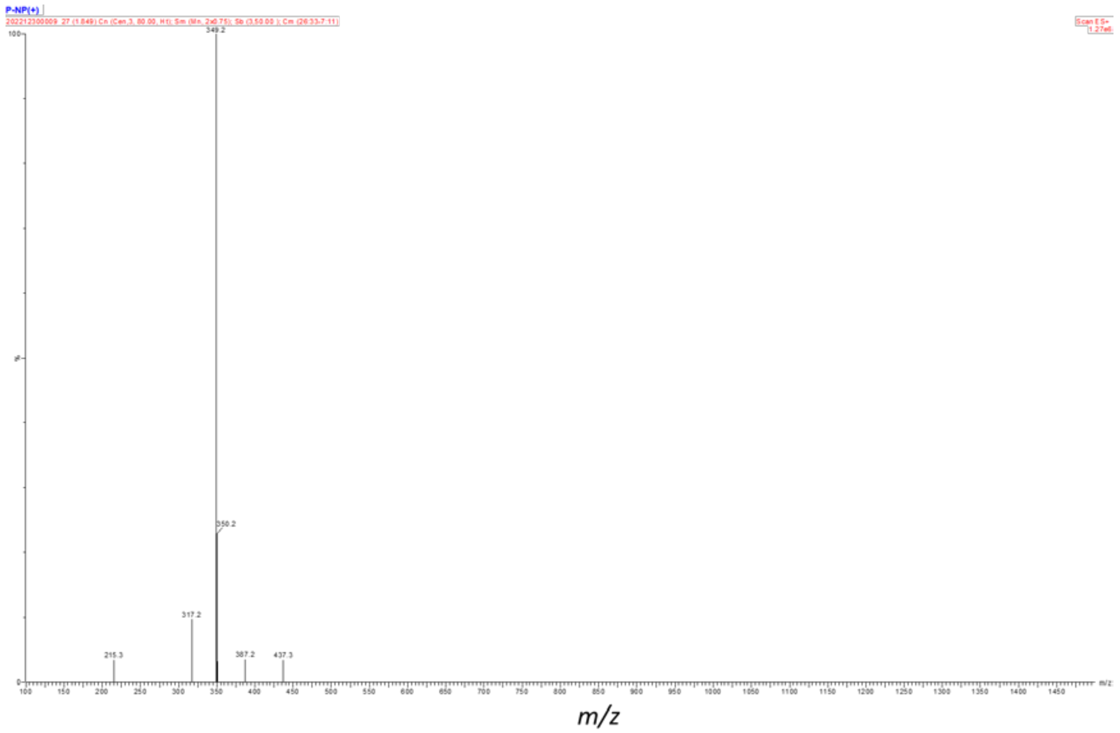

Figure S7. High Resolution-FD-MS spectrum of P-NP.

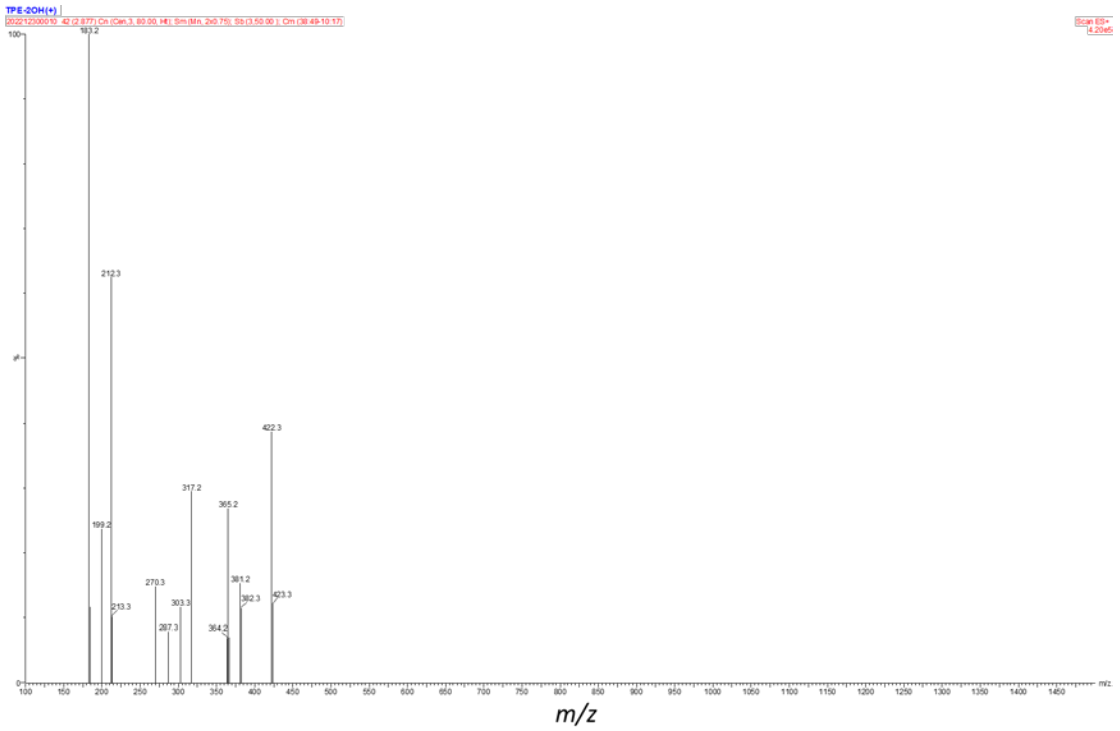

Figure S8. High-Resolution MS spectrum of TPE-2OH.

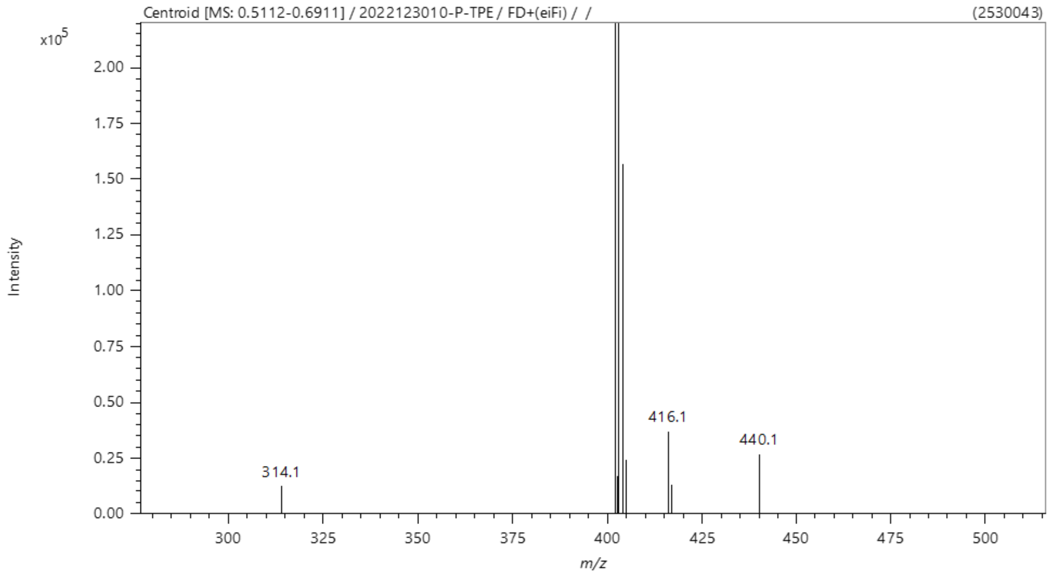

Figure S9. High Resolution-FD-MS spectrum of P-TPE.

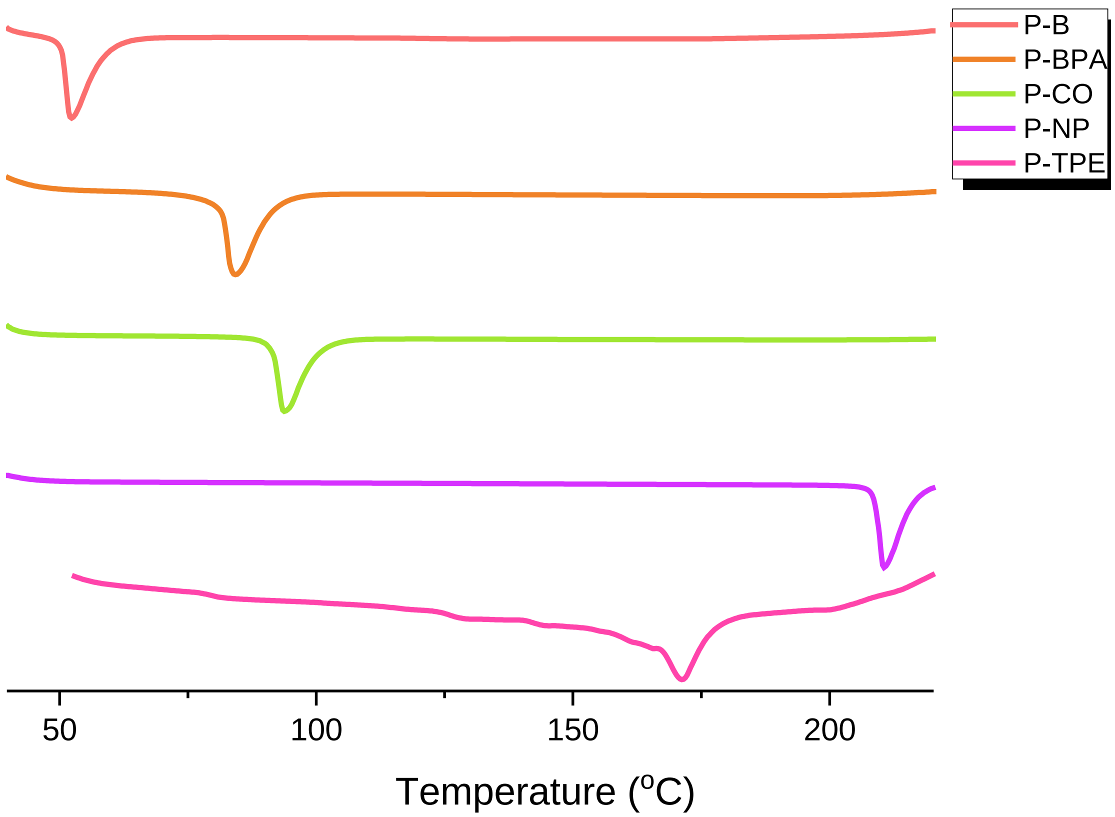

Figure S10. DSC profiles of P-B, P-BPA, P-CO, P-NP, and P-TPE.

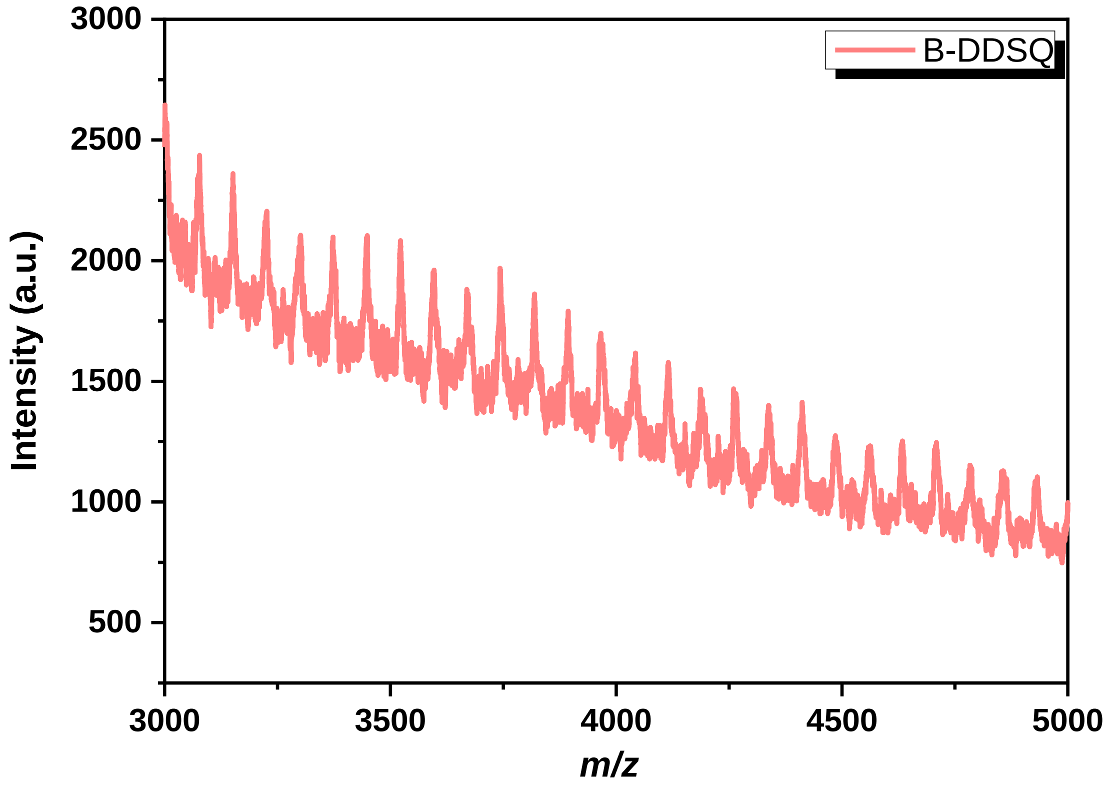

Figure S11. MALDI TOF profile of B-DDSQ.

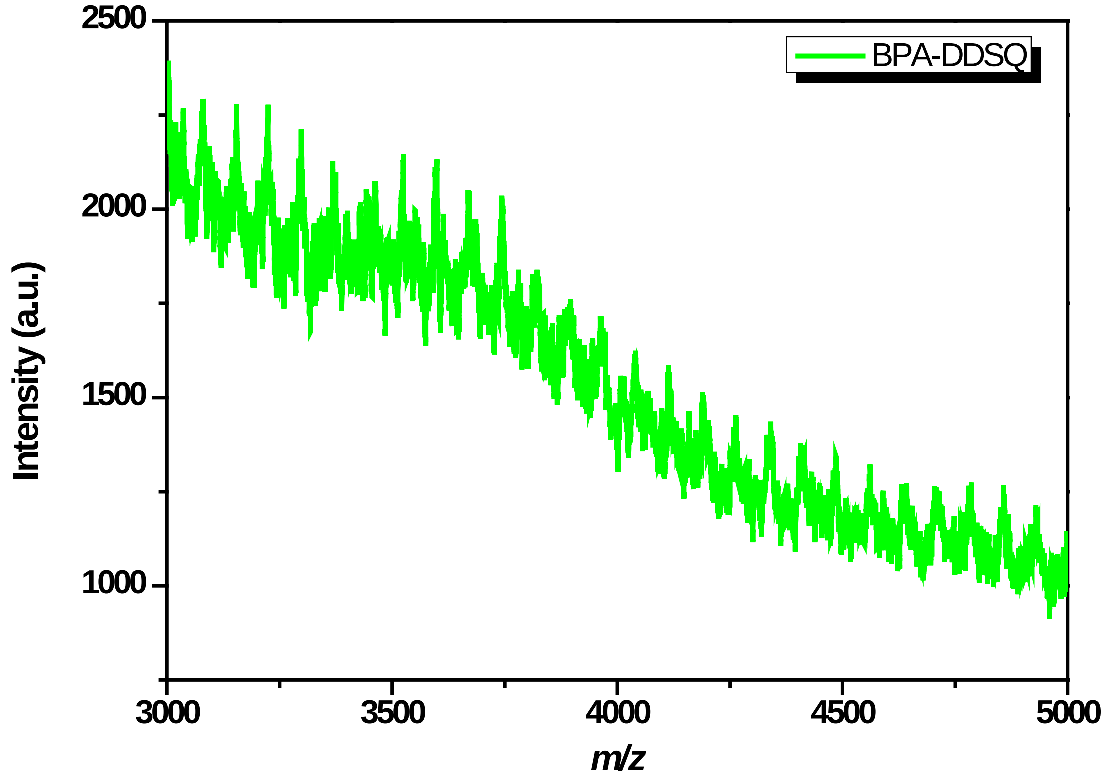

Figure S12. MALDI TOF profile of BPA-DDSQ.

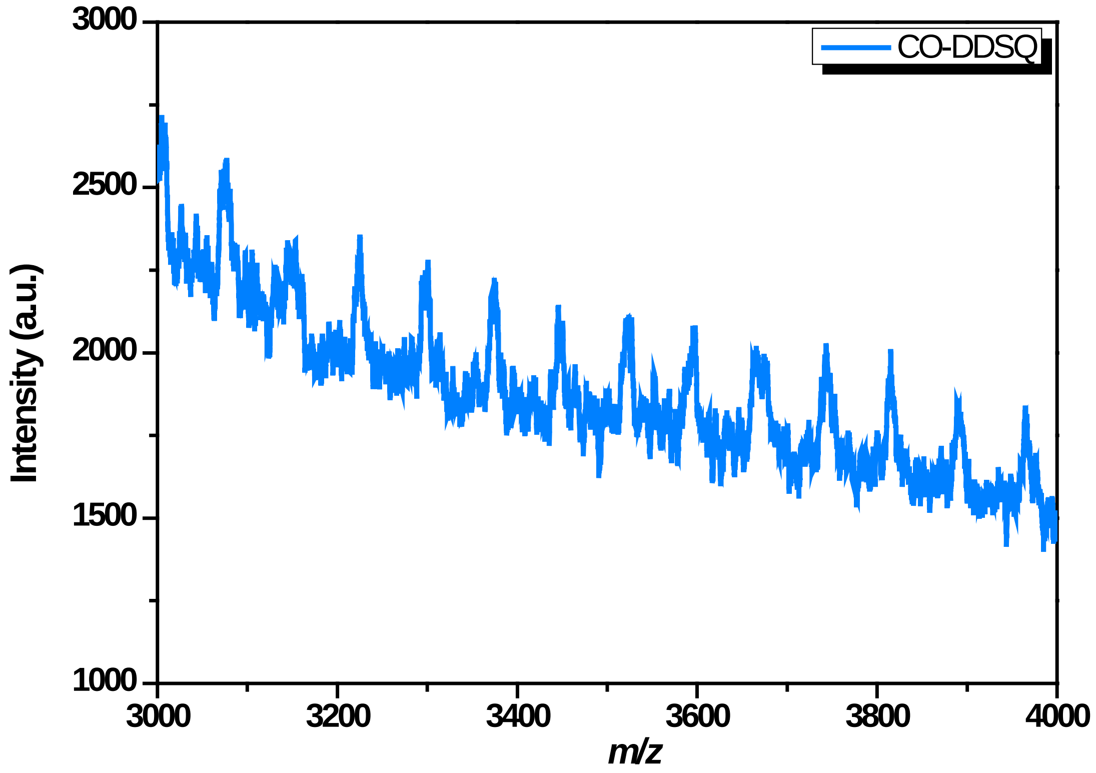

Figure S13. MALDI TOF profile of CO-DDSQ.

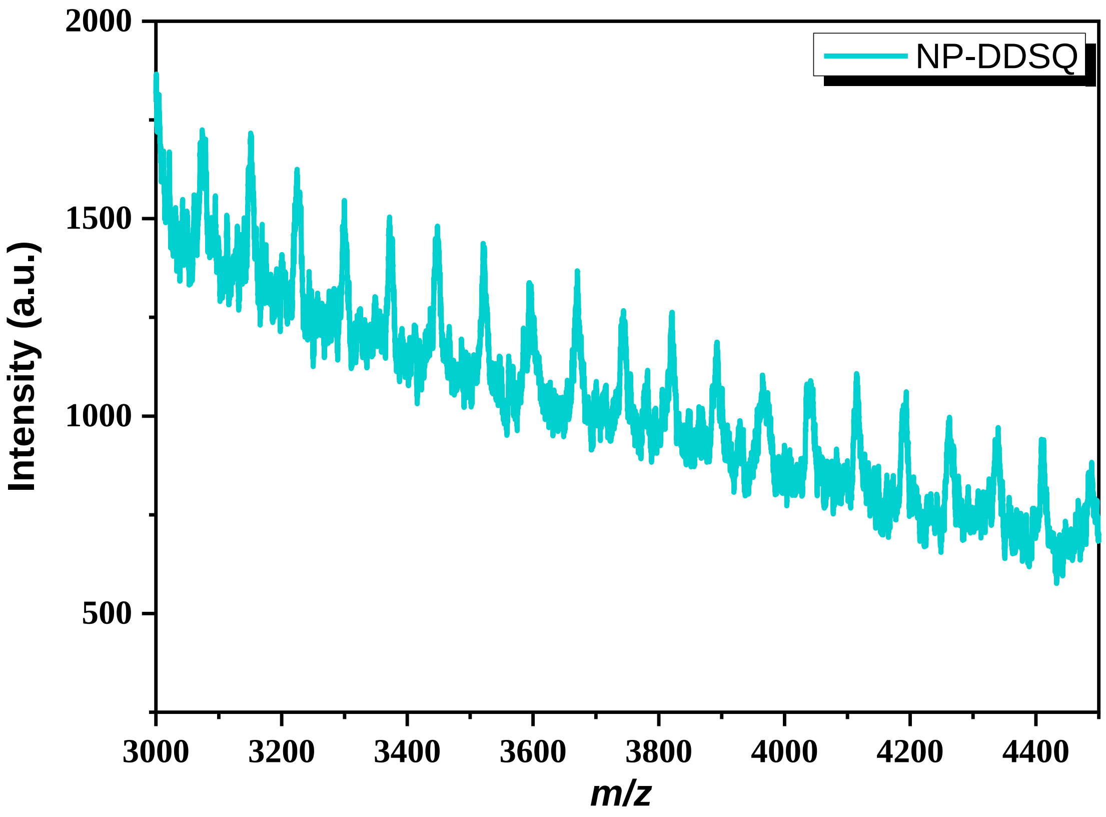

Figure S14. MALDI TOF profile of NP-DDSQ.

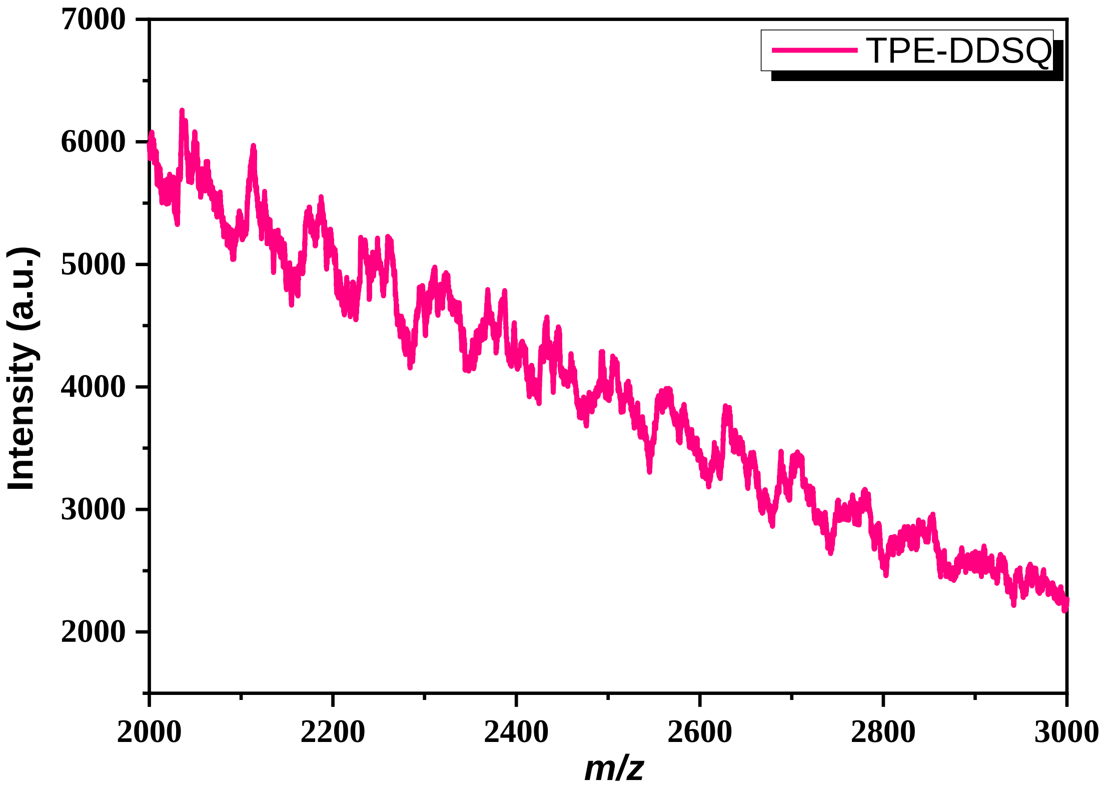

Figure S15. MALDI TOF profile of TPE-DDSQ.

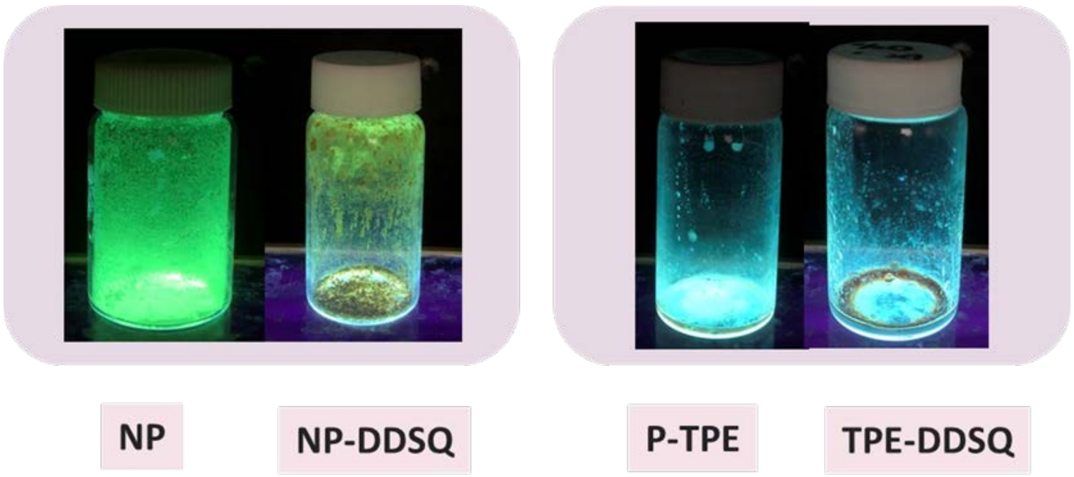

Figure S16. Photo images of NP, P-TPE, NP-DDSQ, and TPE-DDSQ samples under a UV lamp with an excitation wavelength of 365 nm.

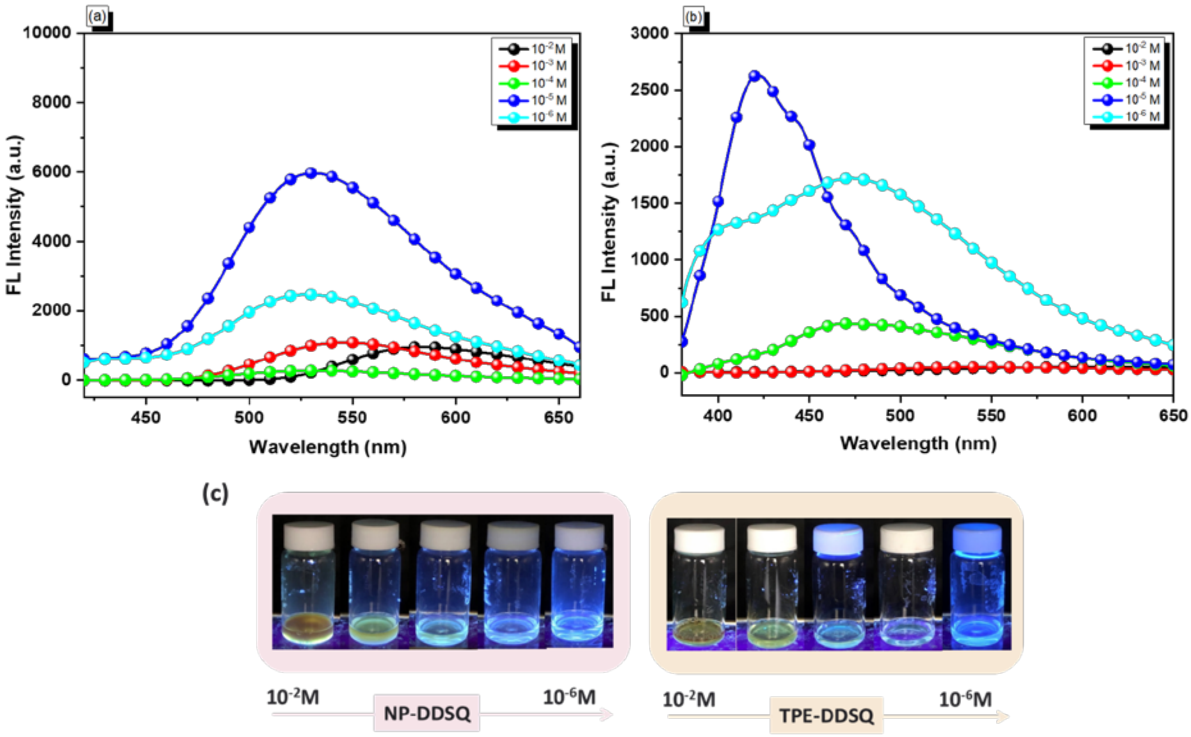

Figure S17. PL spectra of NP-DDSQ (a), (b) TPE-DDSQ, and (c) photo images of NP-DDSQ and TPE-DDSQ in THF solution with different concentrations from 10^{-2} to 10^{-6} M [excitation wavelength was 365 nm].

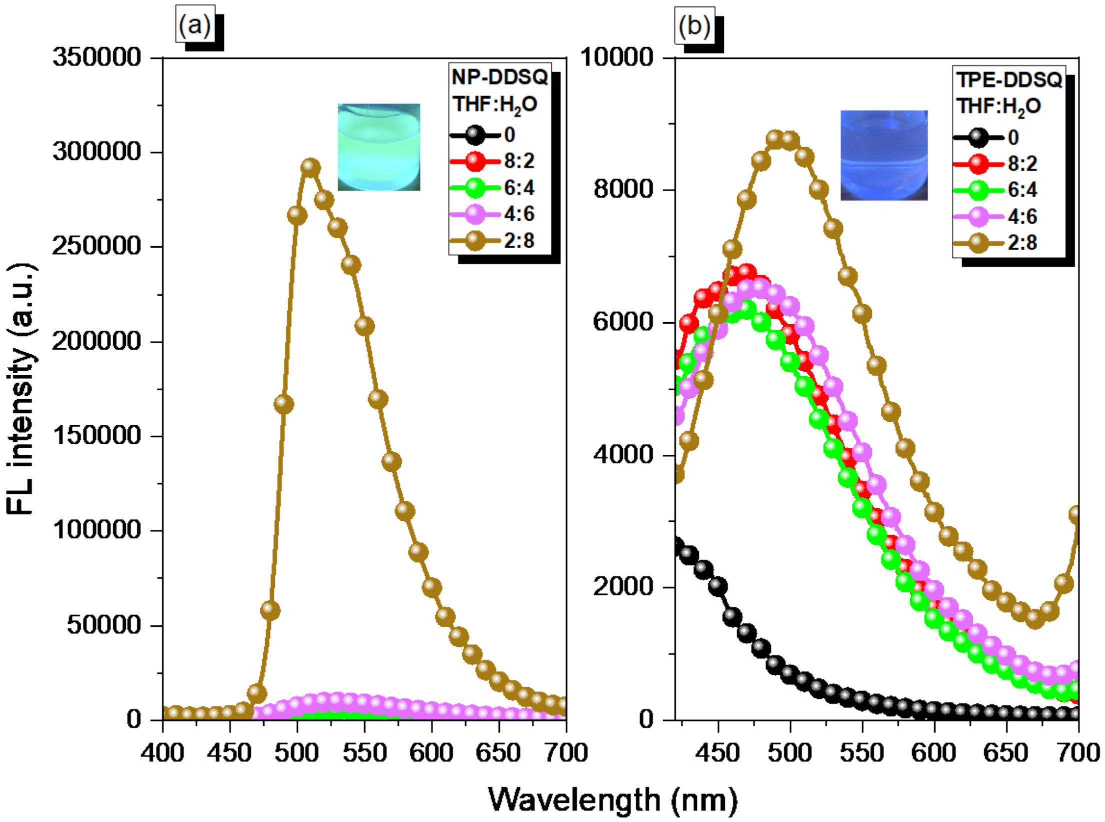

Figure S18. PL spectra of (a) NP-DDSQ and (b) TPE-DDSQ copolymers in THF solution (10^{-5} M) with different water contents [$\lambda_{excitation}$ = 365 nm].

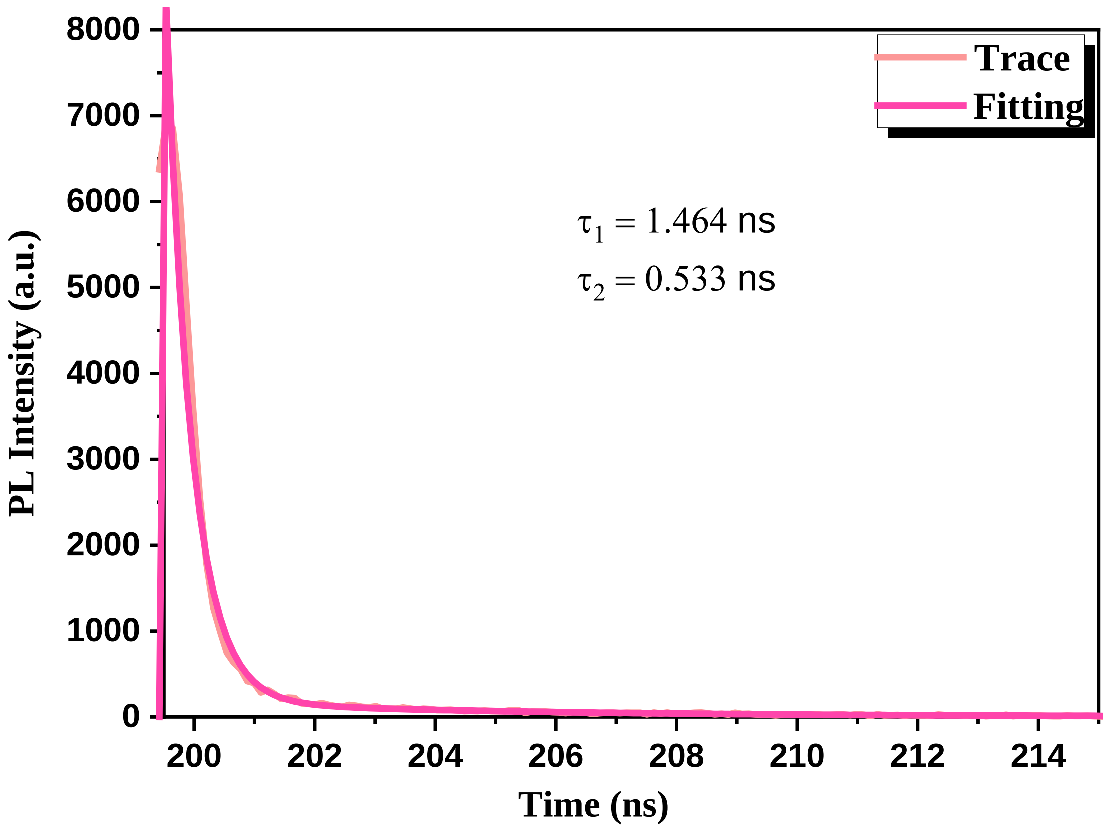

Figure S19. Fluorescence lifetime decay of NP-DDSQ copolymer.

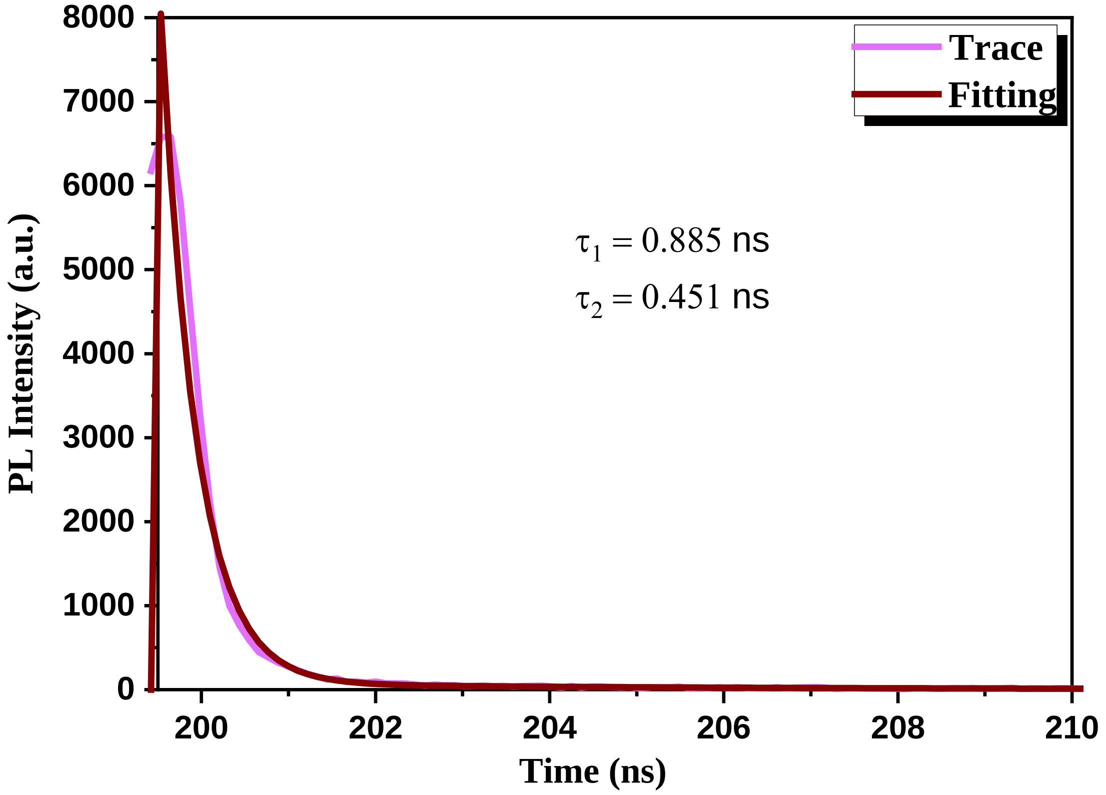

Figure S20. Fluorescence lifetime decay of TPE-DDSQ copolymer.
